# PEO-based brush-type amphiphilic macro-RAFT agents and their assembled polyHIPE monolithic structures for applications in separation science

**DOI:** 10.1038/s41598-017-08423-x

**Published:** 2017-08-10

**Authors:** Aminreza Khodabandeh, R. Dario Arrua, Fotouh R. Mansour, Stuart C. Thickett, Emily F. Hilder

**Affiliations:** 1 Australian Centre for Research on Separation Science (ACROSS), University of Tasmania, Tasmania, Australia; 2 Future Industries Institute, University of South Australia, Building X, Mawson Lakes Campus, GPO Box 2471, Adelaide, SA 5001 Australia; 3 Department of Pharmaceutical Analytical Chemistry, Tanta University, Tanta, Egypt; 4 School of Physical Sciences, University of Tasmania, Private Bag 75, Hobart, 7001 Australia

## Abstract

Polymerized High Internal Phase Emulsions (PolyHIPEs) were prepared using emulsion-templating, stabilized by an amphiphilic diblock copolymer prepared by reversible addition fragmentation chain transfer (RAFT) polymerization. The diblock copolymer consisted of a hydrophilic poly(ethylene glycol) methyl ether acrylate (PEO MA, average Mn 480) segment and a hydrophobic styrene segment, with a trithiocarbonate end-group. These diblock copolymers were the sole emulsifiers used in stabilizing “inverse” (oil-in-water) high internal phase emulsion templates, which upon polymerization resulted in a polyHIPE exhibiting a highly interconnected monolithic structure. The polyHIPEs were characterized by FTIR spectroscopy, BET surface area measurements, SEM, SEM-EDX, and TGA. These materials were subsequently investigated as stationary phase for high-performance liquid chromatography (HPLC) via *in situ* polymerization in a capillary format as a ‘column housing’. Initial separation assessments in reversed-phase (RP) and hydrophilic interaction liquid chromatographic (HILIC) modes have shown that these polyHIPEs are decorated with different microenvironments amongst the voids or domains of the monolithic structure. Chromatographic results suggested the existence of RP/HILIC mixed mode with promising performance for the separation of small molecules.

## Introduction

Macroporous polymer materials with interconnected structures represent a useful class of polymers used in different fields including separation science in the last decades^1^. An increasingly exploited method for the preparation of highly porous scaffolds is based on the solidification of the continuous phase of a high internal phase emulsion (HIPE) through polymerization. A cellular monolithic structure, commonly with interconnected pores and hence an open cellular network is produced, referred to as a poly(HIPE)^2–7^. These materials have been applied extensively to different applications^8^ including membrane separator for batteries^9–12^, electro-chemical sensors^13^, tissue engineering^14–17^, supported catalysis^18^, water purification^19, 20^, and separation science^21–24^. All the demonstrated examples in separation science consist of polymers that are hydrophobic in nature, which limits their applications to separation of non-polar analytes in reversed-phase mode^25^. Introducing polar functional groups in the developed poly(HIPE) makes possible the separation of such analytes of different polarities.

PolyHIPEs with a hydrophilic surface are able to be produced through several different methods: post-synthesis modification of hydrophobic polyHIPEs from water-in-oil (w/o) HIPEs^26–28^, the synthesis of inverse HIPEs (using an oil-in-water (o/w) template) in which the monomer is placed in aqueous phase^29–32^, or the synthesis of bi-continuous hydrophobic polyHIPEs wherein a hydrophilic co-monomer is placed in the aqueous phase of an internal phase in w/o HIPEs^33–36^. Viswanathan *et al*. developed a new method for direct hydrophilic functionalization of a hydrophobic polyHIPE by introducing commercially available polymeric surfactants into a w/o HIPE through physical or chemical entanglement^37^. 3D surface functionalization was obtained in which the hydrophilic part of the polymeric surfactant (such as acrylic acid groups) decorated the surface of the voids of the obtained polyHIPE. Mathieu *et al*. reported the synthesis of a hydrophilic surface modified polyHIPE using an amphiphilic macro-RAFT agent for stabilization of the HIPE template^38^. The presence of RAFT functionality at the chain end of the polymer in the oil phase (styrene and divinylbenzene) provides a possibility for preparation of the porous polymer under RAFT control.

In our previous study^39^, an amphiphilic copolymer (a “macro-RAFT agent”) was used as an anionic emulsifier in an inverse HIPE approach. This method offers attractive possibilities for the development of special coatings of the resultant hydrophilic polyHIPE after the curing step while the RAFT-end group remained at the surface. Our aim is to develop a surfactant-assisted functionalization strategy^38^ for preparation of porous polymers by HIPE polymerization, whereby the obtained porous polymers have a specific application.

**Figure 1:**
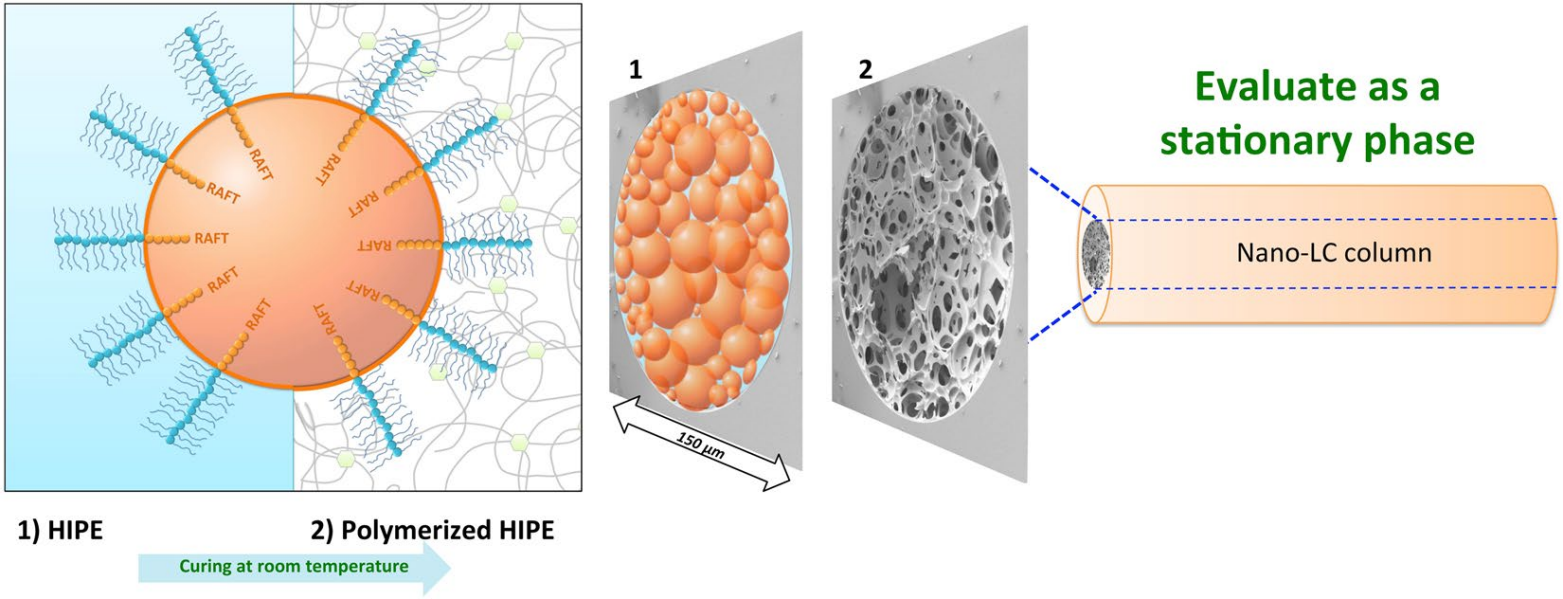
Mechanism of polyHIPE surface functionalization. (**1**) HIPEs stabilized by PEO-based brush-type amphiphilic macro-RAFT agents as surfactants. (**2**) By *in situ* polymerization of the continuous phase, these amphiphilic species can be surface functionalized through PEO brush-type block (physical or chemical) entanglement.

The preparation of a hydrophilic polyHIPE from an o/w HIPE usually requires more careful emulsion stabilization than normal HIPE (w/o HIPE)^40^. The use of PEO-based “brush-like” monomers is anticipated to increase stabilization due to a larger surface area occupied per chain and the higher surface mobility of PEO chains^41, 42^. PEO has been found to provide surfaces with anti-fouling properties as a result of its hydrophilicity, high surface water mobility and low interfacial free energy with water^43^. PEO-based macromolecules have demonstrated their unique potential as steric stabilizers for emulsion polymerization and may enhance their stability against freeze-thaw or shear force^44^. We hypothesize that the PEO-based brush-type amphiphilic macro-RAFT agents with appropriate wettability will be adsorbed at the toluene–water interface, in a similar fashion as polymeric surfactant, and will provide stability against coalescence of the oil droplets, while the PEO block anchoring assists the attachment of these polymeric surfactants to the surface of the obtained polyHIPE upon polymerization (Fig. 1).

As these polymers adopt the format of the mold used as the reactor, an inverse high internal phase emulsion can be introduced into capillary tubing and by *in situ* polymerization of the continuous phase, it can be covalently attached to a surface modified silica capillary. Due to the aspect ratio of the capillary, the morphology of the hydrophilic polyHIPE is likely to differ to that of the bulk material, representing a synthetic challenge to replicate ideal conditions to prepare a porous monolithic structure. These monolithic columns can potentially offer several advantages in the design of high performance columns to be used in liquid chromatography including the high porosity and consequently a low resistance to the mass transfer (low C-term in the van Deemter equation)^45^. In addition, the active chain end (the RAFT-end group) sits at the surface of the material, and its role can be readily studied with respect to potential further surface functionalization.

In this work, the surface chemistry of a hydrophilic polyHIPE inside a capillary format was studied by liquid chromatography. This technique was particularly informative, revealing the role and relevance of the surface chemistry of the polyHIPE with respect to the retention time of different compounds in different modes of chromatography.

## Results and Discussion

### Typical synthesis of the amphiphilic quasi-block macro-RAFT agent

The increasing importance and interest in macro-RAFT block copolymers arise mainly from their unique amphiphilic properties in solution, which are a direct consequence of their molecular structure and presence of the RAFT-end group^38, 39, 46^. While surfactants are selected mostly on trial and error basis for preparation of HIPEs, the hydrophilic-lipophilic balance (HLB) can indicate the capability of forming a certain preferred type of an emulsion. In case of HIPEs, surfactants with the HLB values 2–6 are used for water-in-oil and 12–16 for oil-in-water HIPEs^47^. Specifically targeting a high HLB number (HLB ~16), amphiphilic macro-RAFT agents were synthesized to investigate the effect of the length of the P(PEO MA) and P(Sty) of the macro-RAFT agent with regards to the stability of the inverse HIPE. Table 1 shows the characteristic data for the P(PEO MA)-qb-P(Sty) diblock copolymers synthesized in this study. The SEC analyses of four different macro-RAFT agents are illustrated in Figure S1.

**Table 1:** Macro-RAFT agents synthesized in this study.

(PEO MA)_X_-qb-(Sty)_Y_	X (feed) (PEO MA)^a^	Y (feed) (Sty)^a^	M_n, SEC_ (g mol^−1^)^b^	Đ^b^
Qb-1	5	5	2700	1.19
Qb-2	10	10	4000	1.18
Qb-3	20	20	5400	1.20
Qb-4	50	50	8200	1.42

^a^The feed units obtained a theoretical hydrophilic-lipophilic balance (HLB) value for all macro-RAFT agent around 16, determined by the Griffin’s rule: HLB = 20 × Mh hydrophilic part/Mw (hydrophilic part + hydrophobic part), where Mh is the molecular weight of the hydrophilic block and Mw is the molecular weight of the surfactant. ^b^Determined by SEC in THF (Calibration Sty). Detailed polymerization conditions are provided in Table S1.

This one-pot polymerization technique has been utilized to achieve the synthesis of quasi (block-like) copolymers using sequential monomer addition^48, 49^. This approach yields quasi-block copolymers (Qb) when the conversion of monomer in the first step (e.g. PEO MA) is lower than 100% prior to a second monomer being incorporated. The low dispersity (Đ) of macro-RAFT agent Qb-1 to Qb-3 highlights the RAFT control over the polymerization. These results confirmed that shorter chain length of PEO MA macro-RAFT agents provide high reinitiation efficiency for the polymerization of Sty, as expected based on a previous report^50^.

### Stability of oil-in-water HIPEs using PEO-based macro-RAFT agent

**Figure 2:**
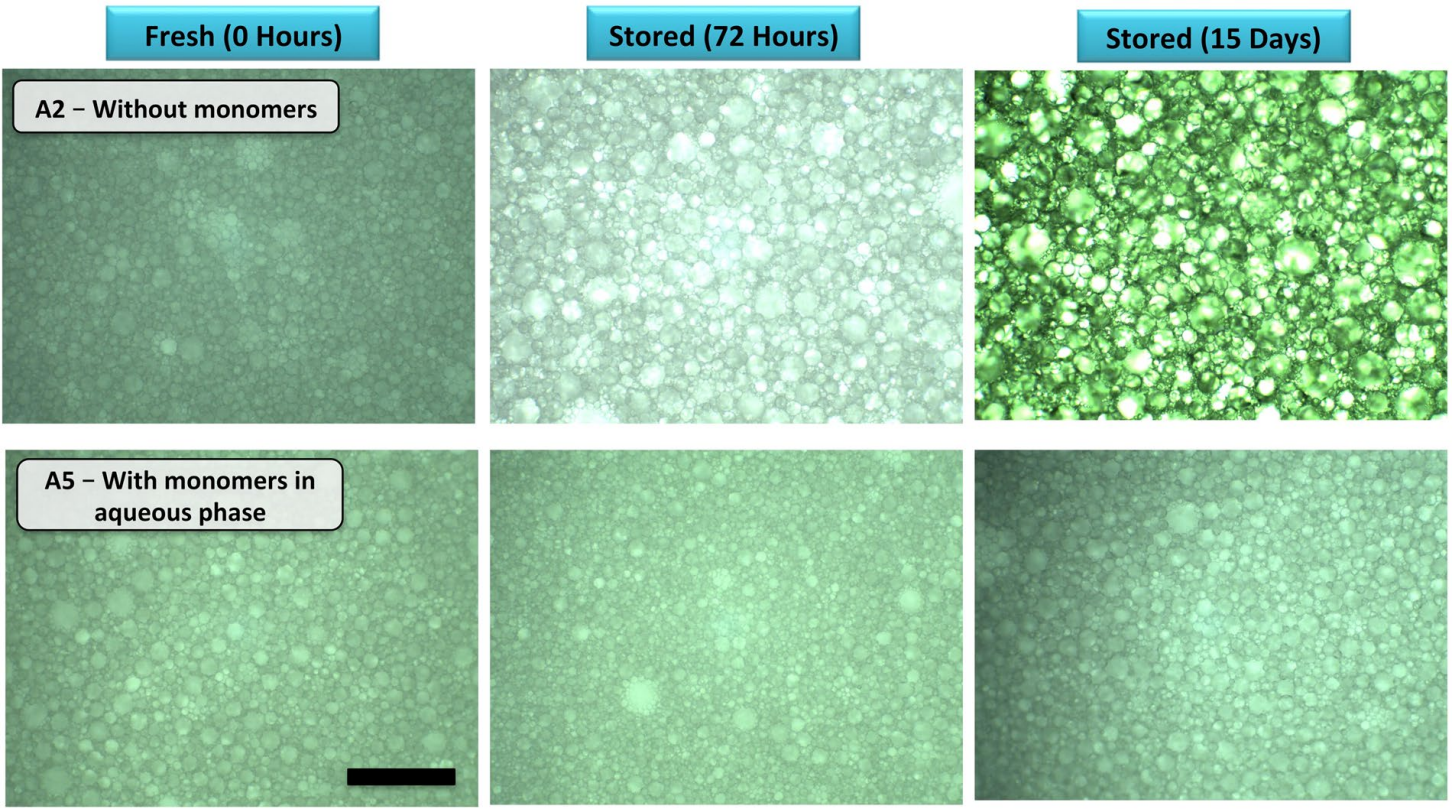
Optical microscopy of HIPEs stabilized by 10 wt% of macro-RAFT agent-Qb2 (w.r.t. the continuous phase); after preparation (0 hours), after 72 hours and after 15 days (right column (HIPE A5)) has AAM and MBAM monomers in the aqueous phase). The scale bar in all cases is 600 μm.

The poly(PEO MA-qb-Sty) quasi-block copolymers prepared here are amphiphilc and can exhibit properties similar to a polymeric surfactant^51^. As a starting point, macro-RAFT agent Qb-2 was chosen (Table 2). The use of 10 wt% of macro-RAFT agent Qb-2 resulted in the successful stabilization of HIPEs with aqueous volume fractions between 60 and 90%. The emulsion droplets were spherical but polydisperse (See Fig. 2 and Figure S2). The drop test method indicated that the HIPE is the inverse system (o/w type)^52, 53^ (see Supporting Information Figure S3).

**Table 2:** Conditions used for the preparation of inverse HIPEs.

Sample code	macro-RAFT agent	wt^a^%	Monomers in aqueous phase	HIPE stability (hours)
A1	Qb-1	10	—	>12
A2	Qb-2	10	—	>15 days ^c^
A3	Qb-3	10	—	>12
A4	Qb-4	10	—	>1
A5	Qb-2	10	AAM-MBAM^d^	>72
A6	Qb-2	20	AAM-MBAM^d^	>72
A7	Qb-2	50	AAM-MBAM^d^	>72
A8	Qb-2	10	AAM-MBAM – 20 wt% more monomers^e^	>24
B1	RAFT-end group removed Qb-2	10	AAM-MBAM − 20 wt% more monomers^e^	>24

^a^All amounts are based on the weight percentage (w.r.t. the continuous phase). ^b^The surface of capillary was modified with 3-(trimethoxysilyl)propyl methacrylate (γ-MAPS). ^c^This HIPE was stable for more than 15 days after preparation. ^d^Monomers in aqueous phase are acrylamide (AAM) and N,N'-methylenebisacrylamide (MBAM). ^e^The amount of the monomers is increased by 20 wt% respect to HIPE A5. For information of voids and windows size see the Table S2.

Preparation of a stable HIPE requires rapid adsorption of the stabilizer at the oil-water interface to lower the interfacial tension between the phases and form a rigid interfacial film^54^. To study emulsion stability, the effect of the number of hydrophilic and hydrophobic units of the polymeric surfactant was investigated using macro-RAFT agents Qb-1 to Qb-4 (see Supporting Information Figure S2). The macro-RAFT agent Qb-2 proved to be sufficiently hydrophilic to stabilize o/w HIPE at least for two weeks (see Fig. 2 and Supporting Information Figure S4). This long-term stability implied that this surfactant was able to suppress the coalescence and Ostwald ripening of emulsion droplets and thus, Qb-2 was selected for further studies. It is also important to mention that the absence of the PSty block (i.e. using a single block RAFT- (PEO MA)_10_ homopolymer as sole emulsifier) resulted in rapidly unstable emulsions, demonstrating the importance of the amphiphilic nature of the stabilizer.

### Synthesis of hydrophilic polyHIPEs

**Figure 3:**
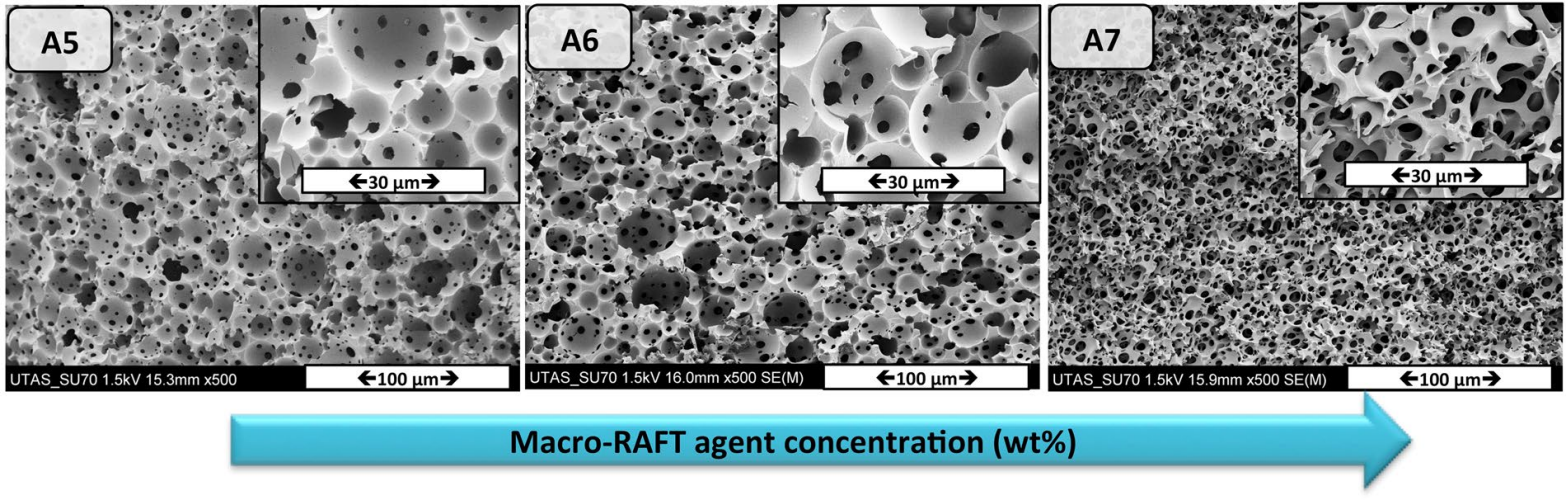
Scanning electron micrographs of emulsion templated macroporous polymer made by polymerization of HIPEs stabilized solely by different amount of macro-RAFT agent-Qb2 (10, 20 and 50 w.r.t. the continuous phase from left to right), polymerized at room temperature (KPS/ TEMED).

A rapid curing of a HIPE system typically locks the emulsion against Ostwald ripening and coalescence, resulting in a homogeneous polyHIPE structure. Inverse HIPEs discussed in the previous section were polymerized using a redox initiation system “potassium persulfate (KPS) /N,N,N',N'- tetramethylethylenediamine (TEMED)” to obtain porous polyHIPEs. PolyHIPE A5 was obtained by using 10 wt% of macro-RAFT agent Qb-2, which retained the shape and volume of the mold. Increasing the macro-RAFT agent-Qb2 concentration from 10 wt% to 50 wt% had a significant effect on the morphology of the resulting polyHIPEs (e.g. on the void size ref. 55) as can be seen from the scanning electron microscopy (SEM) images (Fig. 3). From the SEM images, the number of “windows” per void is increasing from polyHIPE A5 to A7. A higher degree of openness is an advantage for polyHIPEs used in flow-through applications, as it decreases the backpressure of the column once the polyHIPE is introduced to a column housing.

**Figure 4:**
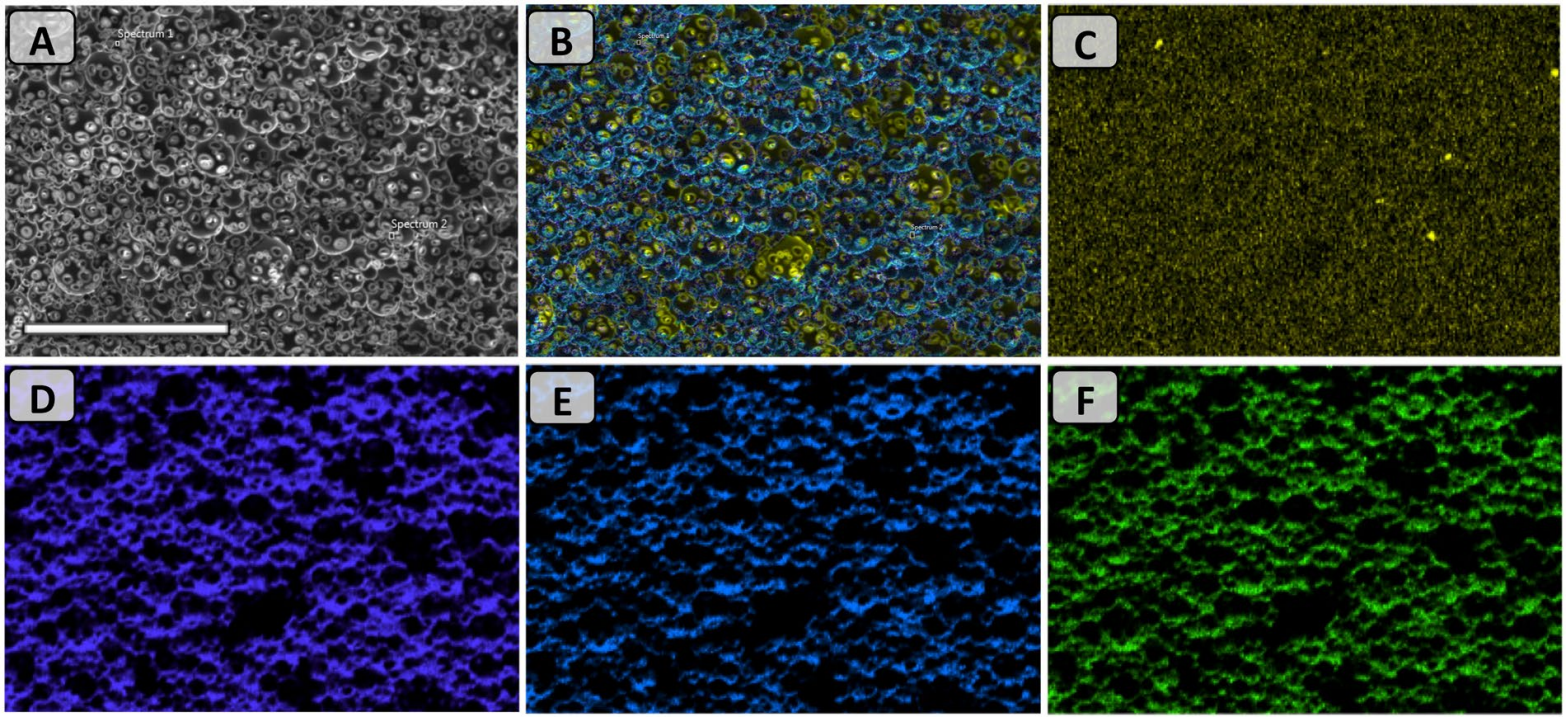
EDX mapping analysis on polyHIPE A5; (**A**) SEM image and (**B**) Overall mapping elements on the same spot: corresponding to sulfur (**C**), carbon (**D**), nitrogen (**E**), and oxygen (**F**) mapping. Scale bar is 100 μm.

The prepared polyHIPEs retained their yellow color after washing process due to the trithiocarbonate group of the RAFT agent. Elemental analysis confirmed the amount of sulfur within the polyHIPEs (e.g. the sulfur content within polyHIPE A5 was 0.43%). Further evidence for the presence of the macro-RAFT agent on the surface of the polyHIPE was obtained from Energy Dispersive X-ray analysis (EDX), clearly indicating that sulfur was present at the surface of the polyHIPE A5 (Fig. 4).

**Figure 5:**
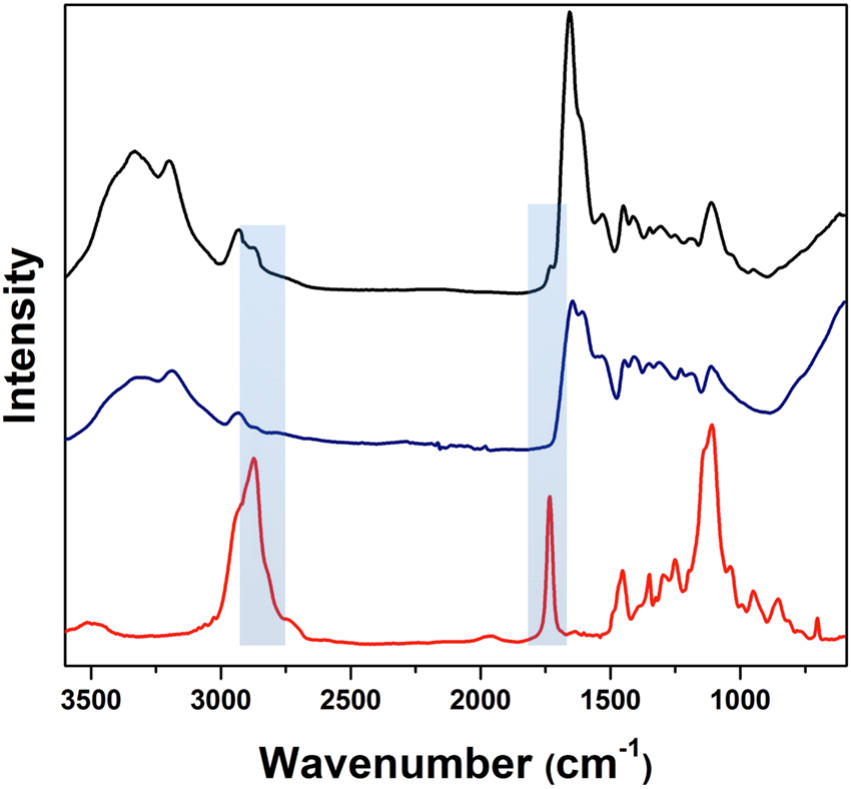
ATR-IR of macro-RAFT agent-Qb2 (red), bulk polymer (blue) and polyHIPE A5 (black). The peaks around 1700–1750 cm^−1^ (related to the C = O stretching of the ester group of the poly(PEO MA) and 2700–2900 cm^−1^ (related to aromatic = C-H stretching of poly(styrene)) are highlighted.

To further investigate the inclusion of the macro-RAFT agent within the polyHIPE structure, Fourier transform infrared spectroscopy (FTIR) analyses were performed on the resultant material, in comparison to a sample of AAM-MBAM polymerized in bulk (KPS/ TEMED as initiators) subjected to the same washing protocol. The FTIR spectrum of polyHIPE A5 shows the presence of an extra band at 1710 cm^−1^ with respect to bulk polymer, which is present in the FTIR spectrum of the macro-RAFT agent (Fig. 5). This signal corresponds to the C = O stretching of the ester group of the PEO MA block, indicating incorporation of the macro-RAFT agent in to the polymer structure. Furthermore, TGA thermograms of the macro-RAFT agent-Qb2, polyHIPE A5 and bulk polymer (see Figure S5 in the Supplementary Information) show similarities in the decomposition profile of polyHIPE A5 and Qb-2, again indicating macro-RAFT incorporation.

**Figure 6:**
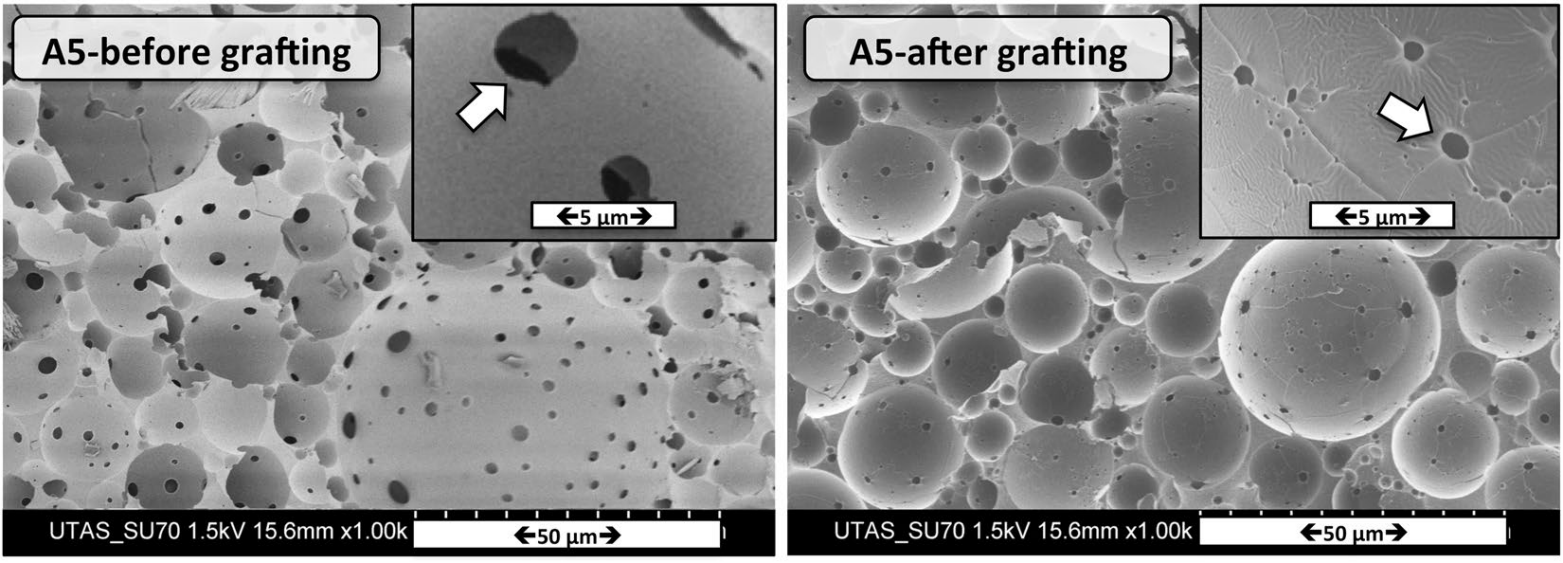
SEM images of poly(HIPE) A5 after “grafting from” polymerization of (4-vinylphenyl)boronic acid.

Grafting experiments utilizing the RAFT-end groups at the polyHIPE surface were performed aiming to demonstrate the presence of the reactive RAFT agent on the surface of voids. Adapting a procedure from Barlow *et al*.^56^, polyHIPE A5 was reacted at 60 °C overnight with (4-vinylphenyl) boronic acid (VPBA). Accordingly, a polyHIPE A5 was treated with a degassed solution of VPBA, RAFT agent and the initiator AIBN (molar ratios 100:5:1) in methanol–acetonitrile (volume ratio 50: 50) at 60 °C for 22 hours. FTIR spectroscopy was used to confirm the presence of poly(VPBA) on the surface, via the presence of B–O stretching peaks (see Figure S6, Supplementary Information)^57, 58^. SEM analysis (Fig. 6) demonstrated a change in surface morphology after surface grafting with VPBA where the size of the windows were decreased. These results clearly demonstrate the availability of the trithiocarbonate group (present on the surface of the functionalized polyHIPEs) for further surface modifications by grafting reactions.

### *In situ* synthesis of hydrophilic polyHIPEs inside a capillary format

A capillary column was chosen as the reactor for the design of hydrophilic polyHIPEs to be used as a stationary phase in chromatographic experiments. The surface of the column was chemically modified with γ-MAPS in order to ensure a covalent attachment between the polymer monolith and the walls of the capillary, subsequently ensuring the mobile phase would flow solely through the voids of the monolith. HIPE A5 was introduced to three different ID capillaries (150, 250, and 500 μm). After preparation of the HIPE A5, the HIPE was placed in ice bath for approximately 5 minutes to lower the temperature of the emulsion. As polymerization commences upon addition of TEMED, this stage is critical with respect to retarding the polymerization of the HIPE, providing sufficient time to fill the capillary using nitrogen gas. After the pre-treated capillary was completely filled with the cold HIPE, the capillary was sealed at both ends with rubber stoppers. The sealed capillary was stored in a dark place at room temperature and allowed to react for 24 h.

**Figure 7:**
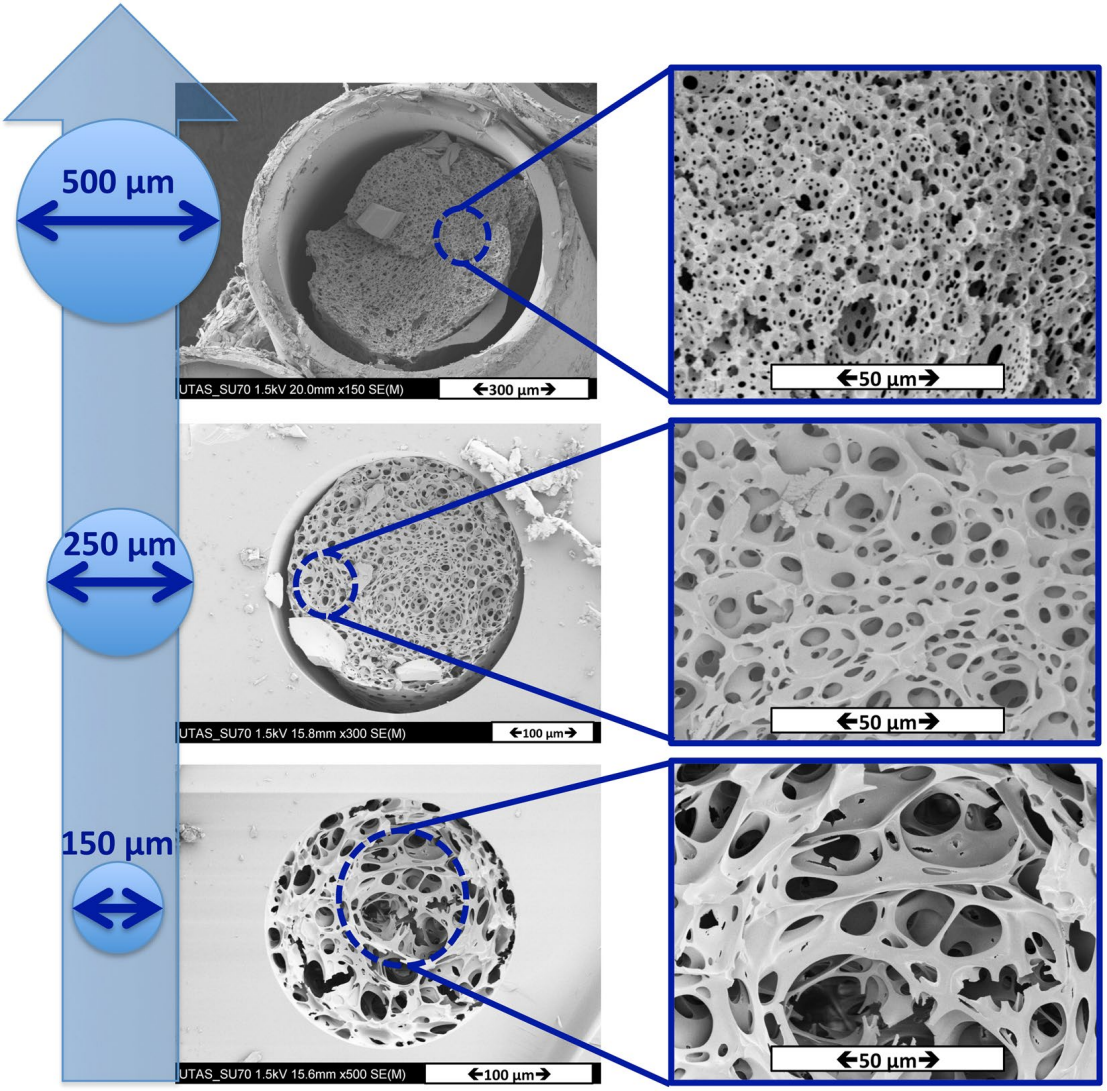
SEM images of poly(HIPE) A5: *In situ* polymerization in different ID capillaries, inner diameters from bottom to top: 150 μm, 250 μm and 500 μm.

As seen in Fig. 7, the morphology of the resulting polyHIPE is strongly dependent on the size of the capillary. The morphology of the polyHIPE in the 500 μm ID capillary is most similar to the bulk structure (see Fig. 3, polyHIPE A5), however there is no attachment to the capillary surface. By decreasing the size of the capillary to 150 μm ID, the polyHIPE structure is attached to the surface but the morphology of the polyHIPE changes significantly. This result may be attributed to the deformability of oil droplets when the column is filling under nitrogen pressure, by considering the increasing likelihood of deformation and or break-up of when the inner diameter is decreased. Furthermore, the extent of polyHIPE shrinkage upon polymerization was studied (see Figure S7 in the Supplementary Information).

**Figure 8:**
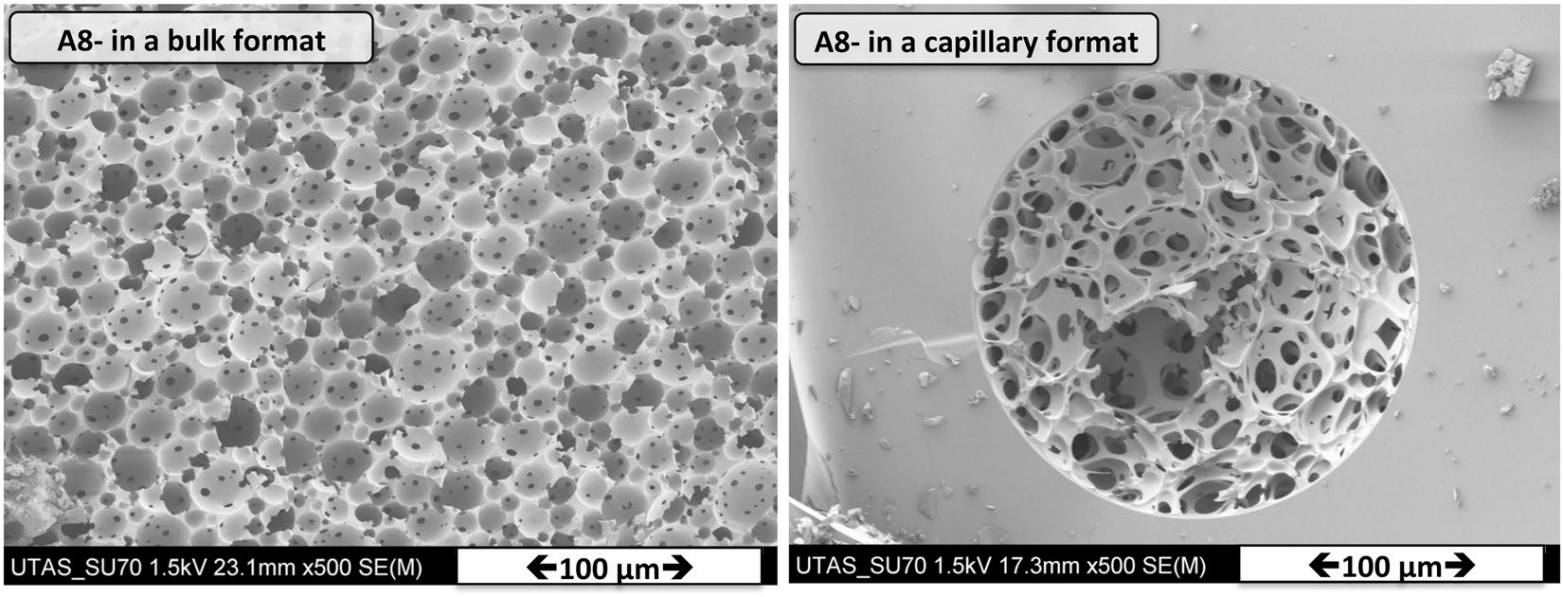
SEM images of poly(HIPE) A8: Polymerization in a bulk (left) and *in situ* polymerization in 150 μm ID capillary (right).

The shrinkage of the polyHIPE structure is ~19% (18.95 ± 4.20%). This is an important factor respect to explaining the de-attachment of the polyHIPE to wall in a larger inner diameter capillary. When the amount of the monomer-crosslinker (AAM-MBAM) in the aqueous phase was increased by 20 wt% (sample A8, Table 2), SEM analysis of the resulting polymer (sample A8, Fig. 8) showed a polyHIPE structure within a capillary housing similar to the bulk morphology.

### Evaluation of the effects of RAFT-end group of the macro-RAFT agent on polyHIPE morphology

We next turned our attention to the role of RAFT-end group of the macro-RAFT agent. It has been reported that the presence of the RAFT-end group in amphiphilic copolymers increases the hydrophobicity of the copolymer^46^. This influences the behavior of a diblock copolymer at an oil-water interface, as it more closely resembles and acts as triblock copolymer.

**Figure 9:**
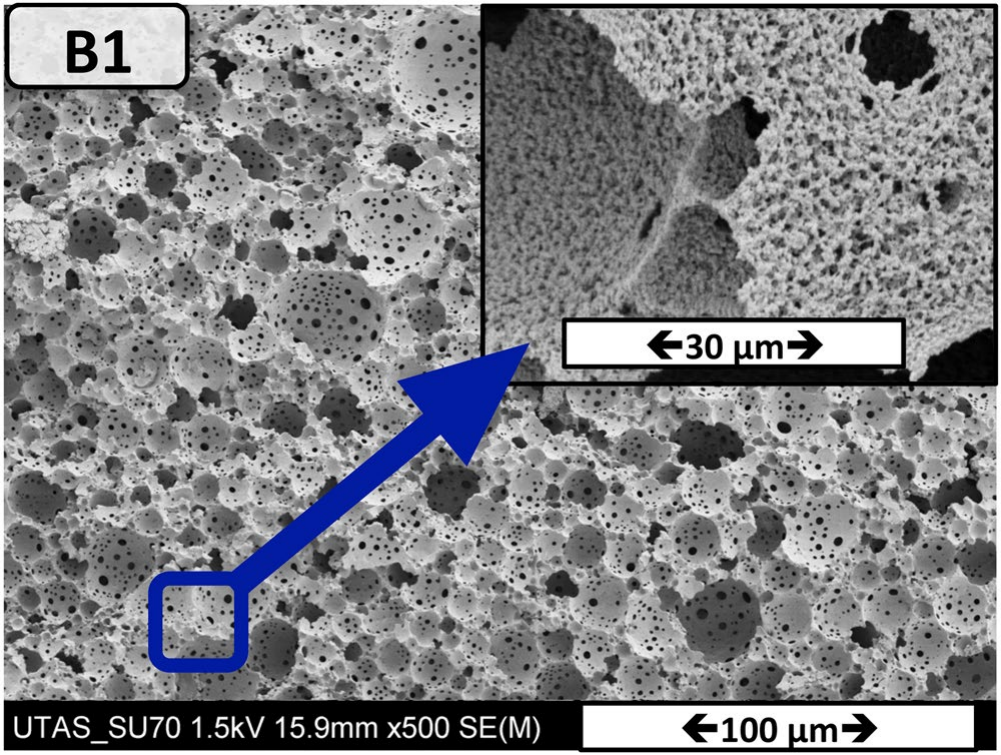
Scanning electron micrographs of emulsion templated macroporous polymer (B1) made by polymerization of HIPEs stabilized solely by end group-removed macro-RAFT agent-Qb2, polymerized at room temperature (KPS/TEMED).

To investigate this, the RAFT part of the macro-RAFT agent Qb-2 (See Table 1) was cleaved using a typical end group removing protocol with minor modifications (see Supplementary Information)^59^. Using this copolymer as a sole stabilizer, a stable inverse HIPE (toluene in water) was obtained. The stability of the HIPE stabilizing by end group removed Qb-2 was investigated by optical microscopy. It was found that both the toluene droplet size and the morphology of the obtained polyHIPE changed. An SEM image of the obtained polyHIPE is shown in Fig. 9. In comparison to poyHIPE A8 (Fig. 8), Fig. 9 shows that polyHIPE B1 possess a hierarchical polyHIPE structure with an increased number of windows. This increased level of interconnectivity was demonstrated with three-fold increase in BET specific surface area (6.75 m^2^g^−1^ for B2, as opposed to 2.07 m^2^g^−1^ for B1).

Our experience with hydrophilic polyHIPEs produced via inverse HIPEs stabilized by Tween 85 (a commercially available, non-ionic surfactant) with paraffin oil as the dispersed phase showed that there is no attachment of this polyHIPE to the modified walls of a capillary format column. Similarly, no attachment of the polyHIPE B1 to the surface of the column was observed in a 150 μm ID capillary (see Figure S8 in the supplementary information). The procedure was repeated in triplicate. The main difference between HIPE B1 and A8 is the presence (or not) of the trithiocarbonate group in the stabilizer used. These results suggest that an end-group removed RAFT copolymer will favor the formation of a hierarchically structured polyHIPE with no attachment to the capillary format column, in comparison to the copolymer cotianing RAFT-end group which enabled full attachment of the polyHIPE to the capillary wall. We believe that the RAFT-end group of the macro-RAFT agent group assures that the monolith is covalently adhered to the capillary (as the polyHIPE is remained attached to the wall after washing with a high pressure), guaranteeing the flow of liquid through the synthesized monolith. Upon removing the butyltrithiocarbonate endgroup, the anchor is changed in the way that the attachment to the capillary wall is not provided.

### Evaluation of hydrophilic polyHIPE as stationary phases in HPLC

**Figure 10:**
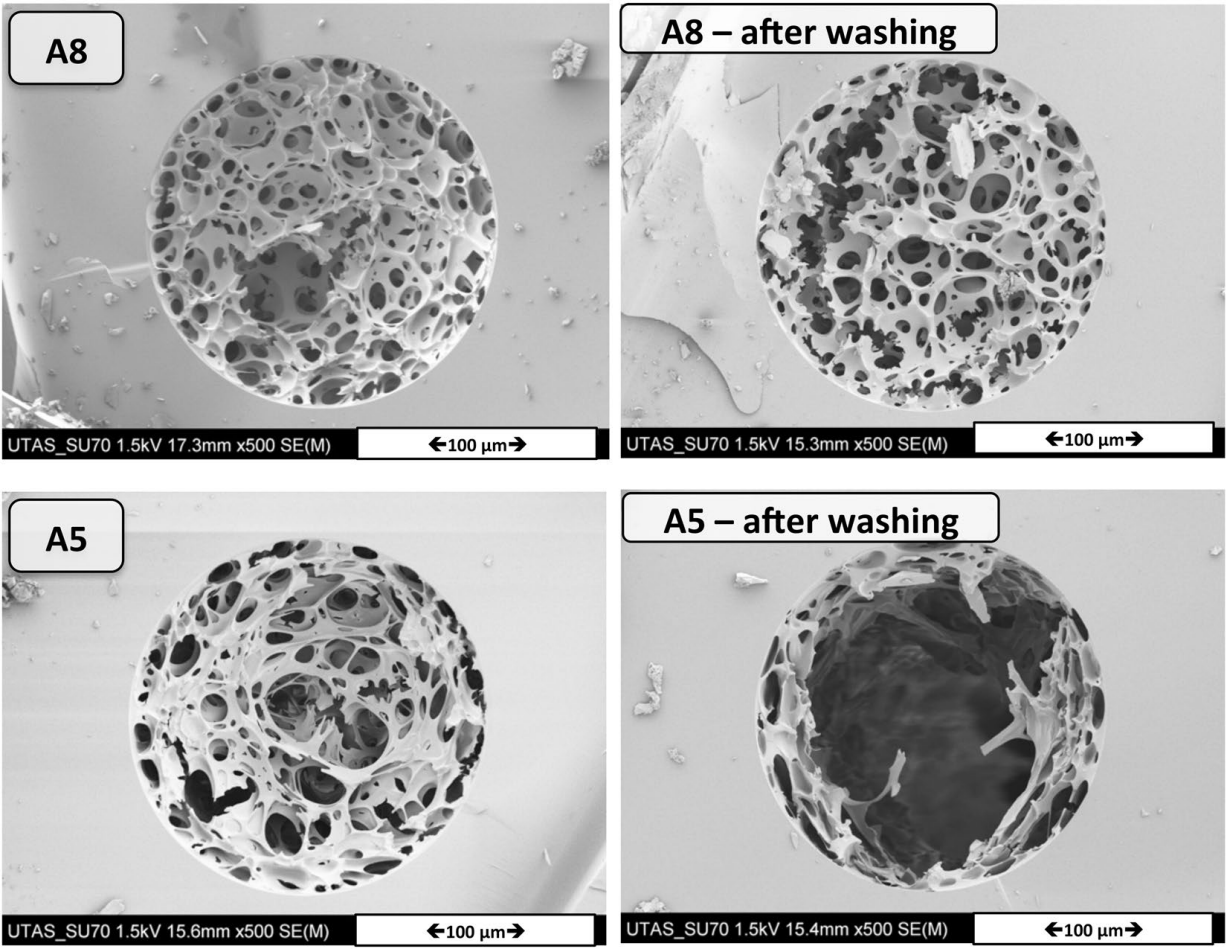
After washing of polyHIPE with water using nano-LC HPLC system.

These as-prepared polymer monoliths in a capillary housing were then evaluated as stationary phases for nano-liquid chromatography. Interactions between analytes (with different polarity) and the polyHIPEs could give us information about the different microenvironments present on the polymer surface. Two capillary columns containing polyHIPE A5 or A8 were studied. The suitability of the polyHIPE structure monoliths was assessed by measuring the backpressure of the materials at different flow rates. The backpressures obtained when both non-swelling (acetonitrile) and swelling (MiliQ-water) solvents were pumped through the polymeric monolith A8 shown in Figure S9. Due to the poor mechanical stability of the polyHIPE A5, it is unsuitable as a stationary phase and this sample was not investigated further (Fig. 10).

Considering the presence of amphiphilic copolymers on the surface of polyHIPEs, the materials are expected to allow the separation of both polar and non-polar analytes. The styrene part in the prepared polyHIPE induces hydrophobic interactions with nonpolar analytes while the surface coverage with PEO MA helps to retain polar analytes. The mechanism of chromatographic retention was studied using two different classes of compounds: non-polar alkylbenzenes to test for the reversed-phase (RP) mode and polar hydroxybenzoic acids to test for the aqueous normal-phase in hydrophilic interaction liquid chromatography (HILIC). Upon injecting the alkylbenzene mixture, the following elution order was observed:

$$\mathrm{Tolene} < \mathrm{ethylbenzene} < \mathrm{propylbenzene} < \mathrm{butylbenzene} < \mathrm{pentylbenzene}$$

**Figure 11:**
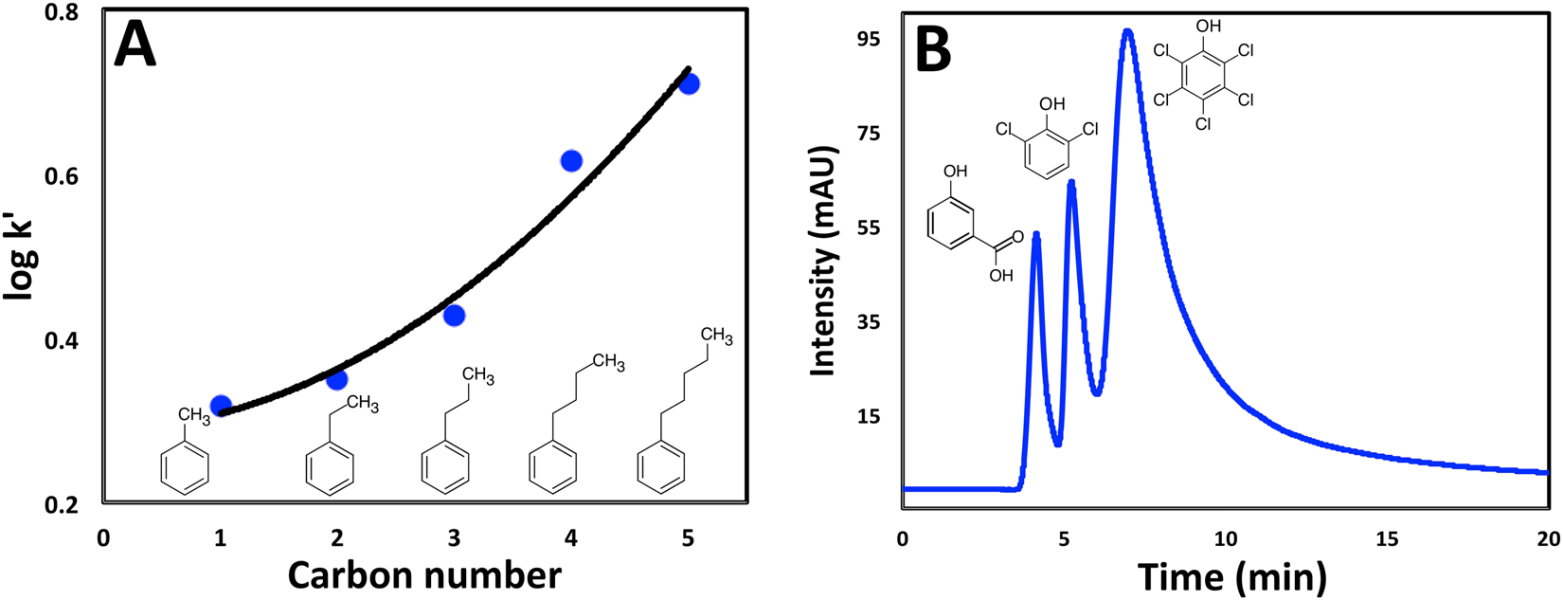
(**A**) Methylene selectivity of the benzene derivatives (toluene, ethylbenzene, propylbenzene, butylbenzene, Pentylbenzene). (**B**) Separation of small molecules in a mixture, from left to right: 3-hydroxybenzoic acid, 2,6-dichlorophenol and pentachlorophenol.

Although this order is typical for reversed-phase mode, the relationship between the length of the aliphatic chain (nc) and the logarithm of the retention factor was nonlinear as shown in Fig. 11-A. This can be explained if another mechanism is contributing to the retention. To investigate further, a mixture of 3-hydroxybenzoic acid, 3,5 dihydroxybenzoic acid, and 3,4,5 trihydroxybenzoic acid was injected. Surprisingly, the least polar analyte; 3-hydroxybenzoic acid was the first to elute followed by 3,5 dihdroxybenzoic acid, followed by 3,4,5 trihydroxybenzoic acid which is the most polar. This order clarifies that HILIC is also involved in the separation process.

It is also worth mentioning that uracil, which normally elutes unretained in the reversed-phase mode was retained to a greater extent than 3-hydroxybenzoic acid, further demonstrating the presence of hydrophilic interactions between polar analytes and the PEO patches on the stationary phase. To determine the predominant mode, the effect of mass fraction of acetonitrile (%ACN) in the mobile phase on the retention time was studied using toluene and 3-hydroxybenzoic acid; an inconsistent change in the retention time was obtained when %ACN was increased which again indicates the existence of RP/HILIC mixed mode. It is important to mention here that the elution order of the two analytes was reversed at high %ACN. That means reversed-phase was dominant at low %ACN while HILIC was predominant at high %ACN. The mechanical stability and efficiency of the column were also studied. The column was stable to the increasing flow rates up to 3.0 μL min^−1^ using aqueous and organic mobile phases. This high permeability enables for increasing the column length and allows for further modification of the column.

As an example of applying this material to the separation of small molecules, a mixture of three analytes (3-hydroxybenzoic acid, 2,6-dichlorophenol and pentachlorophenol) was injected using 20%ACN. As shown in Fig. 11-B, a reasonable separation was obtained which proves that this type of stationary phases could be promising for various applications of chromatographic retention, especially under mixed mode.

## Conclusion

PEO-based, brush-like amphiphilic macro-RAFT agent with a specific HLB value act in the same fashion as common surfactants for the stabilization of oil in water emulsions. As a result, these polymeric stabilizers can be used as a sole stabilizer of an inverse HIPE system to directly prepare hydrophilic polyHIPEs, consisting of cross-linked acrylamide in the continuous phase. The innovative nature of this approach is further illustrated by the high degree of spatial control for placement of functionalities within the monolithic structure. We identified important parameters to take into consideration for *in situ* polymerization of polyHIPE within a capillary column. Furthermore, these columns were investigated as stationary phase for high-performance liquid chromatography. Using a nano-liquid chromatography, it has been shown that the polyHIPE are decorated with different microenvironments amongst the voids or domains of the monolithic structure and the result suggests the existence of RP/HILIC mixed mode with promising performance for separation of small molecules. In addition to the applied context of these materials, this work also serves as the first demonstration of the role of the RAFT group of the emulsifier in the attachment of the obtained polyHIPE to the column surface.

## Experimental Section

### Materials

Poly (ethylene glycol) methyl ether acrylate (PEO MA, average Mn ≈ 480) was purchased from Sigma-Aldrich and used as received. Styrene (Sty, Aldrich, 99%) was passed through a column of Al_2_O_3_ to remove the inhibitor. The RAFT agent, 2-[[(butylsulfanyl)-carbonothioyl]sulfanyl] propanoic acid (PABTC), was synthesized as described in ref. 60. 4,4′-azobis(4-cyanovaleric acid) (V501, >98%, Aldrich) was used as received. Acrylamide (AAM, Sigma-Aldrich, ≥98%), N,N′-methylenebisacrylamide (MBAM, Sigma-Aldrich, ≥99.5%)), methanol (Fluka), basic alumina (Al_2_O_3_, Brockman activity I, 60–325 mesh), N,N,N′,N′- tetramethylethylenediamine (TEMED, Sigma-Aldrich, 99%), were all used as received. 3-(trimethoxysilyl)propyl methacrylate (γ -MAPS) was obtained from Sigma (St. Louis, MO, USA). Toluene was obtained from Chem-Supply (Gillman, SA, AUS). Potassium persulfate (KPS, M&B, 98%) was recrystallized from water.

### Synthesis of PEO-based amphiphilic surfactant by RAFT polymerization

A series of amphiphilic quasi-block macro-RAFT agents (Qb) consisting of PEO MA and Sty were synthesized as reported in the literature^50^. The PEO-based (PEO MA, average *M*_n_ ≈ 480) was selected as it provides a hydrophilic group to assist the solubility of the macro-RAFT agent in the aqueous continuous phase. A typical polymerization protocol that was adopted is summarized: In first step, 1 g (4.20 × 10^−3^ mol) of PABTC and 0.12 g (4.20 × 10^−4^ mol) of V501 were introduced to a round-bottom flask and which was then sealed with a rubber septum, and solids were purged with ultra pure argon for 10 min. In a second step, 10.08 g (2.10 × 10^−2^ mol) of PEO MA was then dissolved in 100 mL of dioxane before addition to the round-bottom flask to obtain a solution. This was purged with ultra pure argon for 10 min. The reaction was allowed to proceed at 70 °C for 6 h under constant stirring. After quenching the reaction in an ice bath, a small aliquot of the solution was removed for ^1^H NMR analysis to determine the conversion of the PEO MA single block. Styrene and V501 were then added to the round bottom flask at a molar ratio (relative to the initial chain transfer agent concentration) equal to the desired number of monomer repeat units per macro-RAFT agent. The mixture was purged with ultra pure argon for 10 min and further polymerization for 12 h at 70 °C was performed. After which a small aliquot of the solution was removed for SEC and^1^H NMR analysis. The degree of polymerization of the macro-RAFT agent was determined by ^1^H NMR spectroscopy. Dioxane was then removed through rotary evaporation under reduced pressure and all polymers were purified by dialysis. Dialysis involved placing the polymer into dialysis tubing (MWCO 2000) and then submerging the polymer and tubing in deionised water (DI) with agitation (see Supporting Information Figure S10). Water was removed via freeze-drying of the macro-RAFT agents at −30 °C under reduced pressure for at least 100 hours. The polymer was then stored at 4 °C until use. Figure S11 shows the NMR spectra of RAFT- PEO MA_m_-b-Sty_n_ (Table 1).

### Synthesis of hydrophilic ‘inverse’ polyHIPEs

The macro-RAFT agent was dissolved in 4 ml water without any adjustment of pH. Toluene (16 ml) was added drop-wise to an aqueous solution of macro-RAFT agent with a desired concentration; at a rate of 0.8 mL min^−1^ with constant stirring at 1000 rpm. The emulsion was stirred for an additional 20 min after complete addition of the internal toluene phase. The drop test method was used to determine the type emulsion prepared and optical microscopy was used to examine emulsion stability.

The macro-RAFT agents prepared were used as stabilizers of o/w emulsions. A range of different monomers and crosslinkers were tested attempting to obtain macroporous polyHIPEs (see Supporting Information Figures S12–S13). A successful monomer and crosslinker couple; acrylamide (AAM, 1.420 g, 1.99 × 10^−2^ mol) and the crosslinker N,N′-Methylenebisacrylamide (MBAM, 0.309 g, 2.00 × 10^−3^ mol) were dissolved in 4 ml of water containing macro-RAFT agents. The initiator KPS (0.04 g, 1.47 × 10^−4^) was also dissolved in the above aqueous solution. The dispersed phase, toluene (16 mL) was then added drop-wise. The emulsion was stirred for an additional 20 min after complete addition of the internal toluene phase. The emulsion was transferred to a mold (a glass container) and appropriate amounts of TEMED were added to emulsion after formation, which already contained KPS and cured at room temperature. The resulting polyHIPE was purified via Soxhlet extraction with methanol for 48 h as well as 48 h with water. The purified monolith was dried under vacuum oven for at least 72 h to constant weight under vacuum at 30 °C. The experimental conditions used for the preparation of the different polyHIPEs can be found in Table 2.

### *In situ* preparation of hydrophilic polyHIPE columns

A capillary format was chosen as a ‘column housing’ for poly(AAM-MBAM) based hydrophilic polyHIPE to be evaluated as stationary phases for nano-LC. Prior to the polymerization, fused silica capillaries with different internal diameters were modified with 3-(trimethoxysilyl)propyl methacrylate using a procedure previously described^61^ (see Supporting Information Figure S14). Using an ice bath to retard the polymerization reaction, an inverse HIPE was introduced to the capillary column using pressure of nitrogen (see Supporting Information Figure S15). *In situ* polymerization of an inverse HIPE in a capillary was conducted using a KPS/TEMED redox couple as initiator.

### Chemical stability and swelling behavior of monolithic columns

The chemical stability of polyHIPEs in capillary formats was described by pressure drop of monolithic columns at different flow rates using pure water and acetonitrile as mobile phase. For each flow rate, the pressure values of the HPLC system were measured without and with the column, and the pressure drop across the monolith was calculated as the difference between these two values.

### Characterization

NMR analyses was performed on a Bruker Ultra Shield Avance Spectrometer (600 MHz). For all NMR analyses deuterated solvents were used as stated. Size exclusion chromatography (SEC) was performed with a Viscotek instrument using refractive index detector (RID) and two chromatography columns (two PSS S linear 3 μm, Polymer Standard Services GmbH, PSS), THF (HPLC grade) was used as an eluent at a flow rate of 0.5 mL/min. The column oven was kept at 40 °C. The calculated molecular weights were based on a calibration curve for polystyrene (PSty) standards of narrow polydispersity with a molecular weight range of 160–154000 g/mol (PSS-Polymer Laboratories). The standards were prepared and injected, the column injection volume was 0.1 mL.

Emulsion droplets were observed by optical microscopy (Nikon, model Eclipse E200), equipped with a camera (Tucsen, model IS500). Images of the emulsions were analyzed by ImageJ (NIH image)^62^. From these, the mean droplet size and size distribution were evaluated. Three samples were analyzed for each experiment and the reported results are the average of these. More than 100 droplets were measured. PolyHIPEs were characterized by field emission gun scanning electron microscopy (FE-SEM) studies using a Hitachi SU-70 FESEM in the Central Science Laboratory, University of Tasmania. All samples were platinum coated for 15 s in an argon atmosphere (Emitech 550, Emitech Ltd., UK). The composition of the material was examined by EDX experiments where the materials were sputter-coated with carbon (Ladd 40000 carbon evaporator) before analysis. The calculation of the average pore and windows diameter was performed on sets of at least 100 pores and 100 windows, respectively, using the image analysis software ImageJ (NIH image). A statistical correction was employed to obtain more accurate value, as each value was multiplied by 2/(3^1/2^) as described by Carnachan *et al*.^63^.

The sulfur content of the polyHIPEs was determined with a Thermo Finnigan EA 1112 Series Flash Elemental Analyser. Thermogravimetric analyses were carried out using Setaram LABSYS Evo TG-DSC Thermogravimeter in the temperature range from 30 to 600 °C at the heating rate of 5 °C min^−1^ under nitrogen atmosphere. The sample mass was about 15 mg. FTIR spectra were recorded by a Bruker Vertex 70 infrared spectrometer equipped with an ATR probe. The Brunauer–Emmett–Teller (BET) surface area and microporosity were assessed using a Tristar II analyzer for the nitrogen adsorption/desorption at 77 K (Particle and Surface Science, Gosford, AUS). The nano-liquid chromatography studies were performed using an Ultimate 3000 RSLCnano system (Dionex, Sunnyvale, CA). A 1μL sample loop was used and the system was operated with Chromeleon software. UV absorbance was monitored at 214 nm.

### Data availability statement

The datasets generated during and/or analysed during the current study are available from the corresponding author on reasonable request.

## Electronic supplementary material

Supplementary info
