# Supplementary material for: PEO-based brush-type amphiphilic macro-RAFT agents and their assembled polyHIPE monolithic structures for applications in separation science
Source: Sci Rep. 2017 Aug 10;7:7847. doi: 10.1038/s41598-017-08423-x (PMC5552774; doi:10.1038/s41598-017-08423-x)
Supplement: Supplementary file 1 — Supplementary info [file 41598_2017_8423_MOESM1_ESM.doc]

Supplementary information for

**PEO-based brush-type amphiphilic macro-RAFT agents and their assembled polyHIPE monolithic structures for applications in separation science**

Aminreza Khodabandeh^1,4^, R. Dario Arrua^2^, Fotouh R. Mansour^1,3^, Stuart C. Thickett^4^ and Emily F. Hilder^2*^

^1^Australian Centre for Research on Separation Science (ACROSS), University of Tasmania, Tasmania, Australia

^2^Future Industries Institute, University of South Australia, Building X, Mawson Lakes Campus, GPO Box 2471, Adelaide SA 5001, Australia

^3^Department of Pharmaceutical Analytical Chemistry, Tanta University, Tanta, Egypt

^4^School of Physical Sciences, University of Tasmania, Private Bag 75, Hobart 7001, Australia

**Tables of Contents**

**S1. Introducing monomers and cross linkers to aqueous phase**

**S2. Typical procedure for surface modification of fused-silica capillary**

**S3. Typical end-group removal process**

**Figure S1.** A) SEC chromatograms of macro-RAFT agents, from right to left: Qb-1 (red), Qb-2 (black), Qb-3 (blue) and Qb-4 (pink).

**Table** **S1.** RAFT synthesis of PEGA-qb-PSty.

**Table S2.** Conditions used for the preparation of inverse HIPEs.

**Figure S2.** Stability of toluene in water HIPE stabilized by macro-RAFT agent. All HIPE with 80% toluene in water solely stabilized with 10 wt % of (from the left to right): macro-RAFT agent Qb1, macro-RAFT agent Qb2, macro-RAFT agent Qb3 and macro-RAFT agent Qb4. No monomers were introduced in the HIPEs.

**Figure S3.** The emulsion type was determined by the drop test method. One drop of the formed HIPE with macro-RAFT agent-Qb2 was placed into (A) water and (B) toluene. The emulsion droplet was seen to disperse in the water but remained as a droplet in toluene.

**Figure S4.** Stability of toluene in water HIPE stabilized by macro-RAFT agent-Qb-2.

**Figure S5.** (A) TGA analysis of macro-RAFT agent-Qb2 (red), polyHIPE A5 (black) and bulk polymer (blue). B) T_max_ data.

**Figure S6.** FTIR spectrum of poly(HIPE) A5 before (black) and after (red) “grafting from” polymerization of (4-vinylphenyl)boronic acid. The peak around 1375–1425 cm^−1^ is highlighted.

**Figure S7.** Shrinkage study on polyHIPE A5.

**Figure S8.** Polymerized HIPE B1 stabilized by end group removed macro-RAFT agent, *in situ* polymerization in 150 μm ID capillary.

**Figure S9.** Back pressure studies on capillary A8, water (red) and acetonitrile (blue).

**Figure S10.** Typical procedure for purification of macro-RAFT agent by using dialysis tubing (MWCO 2000).

**Figure S11**. ^1^HNMR spectra of macro-RAFT agent Qb-2 (DMSO-d6).

**Figure S12.** Monomers and cross-linkers used in aqueous phase.

**Figure S13.** Typical polymerization of monomers in water in presence of redox initiation system (TEMED/ KPS). From the left to right: Glycerol 1,3-diglycerolate diacrylate, Pentaerythritol triacrylate, Tetra(ethylene glycol) diacrylate, Bis[2-(methacryloyloxy)ethyl] phosphate, Poly (ethylene glycol) diacrylate, f) 2-Hydroxyethyl methacrylate, acrylamide.

**Figure S14.** Surface modification of a fused-silica capillary surface using 3-(trimethoxysilyl)propyl methacrylate (γ -MAPS).

**Figure S15.** Using nitrogen pressure to fill a capillary format column with an inverse HIPE.

**S1. Introducing monomers and cross linkers to aqueous phase**

A series of monomers was tested in regard to add to the aqueous phase. It is apparent that monomers should be dissolved in water as well as has a small partition coefficient to the toluene (which is used as template and if monomer tend to droplets, after polymerization some heterogemiety would be expected). All selected monomers are important as obtained polymer used as stationary phase in separation science.

While the stable inverse HIPE is provided a template for preparation inverse HIPE with different monomers, the solubility of monomers in the aqueous phase is limited the choices. This emphasis the importance of developing surfactant-assisted functionalization strategy as tuning the surface chemistry using different functional monomer is limited.

**S2. Typical procedure for surface modification of fused-silica capillary**

Briefly, the silanol groups on the surface of fused-silica monolith were activated by introducing an etching step. Then, the capillary column with a length of 35 cm was rinsed with 0.1 M NaOH for 1 h and then with water until the outflow reached pH 7.0. After subsequent flushing with methanol for about 10 min, it was dried by passage of nitrogen gas. γ-Methacryloxypropyltrimethoxysilane (γ-MAPS) solution by its dilution with methanol (pH= ~4) at a volume ratio of 1:1 was injected into the capillary with a syringe. It was sealed with rubber at both ends and then submerged in a bath of water at 50 °C for overnight. Finally, the capillary was rinsed with methanol and water to flush out the residual reagent.

**S3. Typical end-group removal process**

Typical end-group removal process was applied on macro-RAFT agent Qb-2, using benzoyl peroxide as initiator. Briefly, A mixture of macro-RAFT agent Qb-2 (0.2 g, 0.13 mmol), Benzoyl peroxide (BPO) (0.5 g, 2.06 mmol), and toluene (6 g) was placed in a round-bottom flask, sealed, and degassed with argon gas for 20 minutes. 2-Propanol was degassed with argon gas in a separate sealed round-bottom flask. The 2-propanol (6 g) was removed through a syringe equipped with a long needle and injected to the mixture. The round-bottom flask containing the mixture was then heated to 100 °C for 3 h. The completion of butyl-trithiocarbonate RAFT-end group removal was determined by ^1^H-NMR after evaporating the volatile solvents from the product in a vacuum oven at 40 °C overnight. The ^1^H-NMR spectrum of the product demonstrated the absence of signals associated with the butyl trithiocarbonate end group at 3.3 ppm (CH_3_-(CH_2_)_2_ -CH_2_-S-C(S)-S-) and 4.8 ppm (the first chain length of CH oligomer backbone adjacent to the sulfur). The evaporated product was dissolved in 2-propanol and was purified by precipitation method in a cold methanol/water mixture (80/20 v/v %) to remove the unreacted BPO.


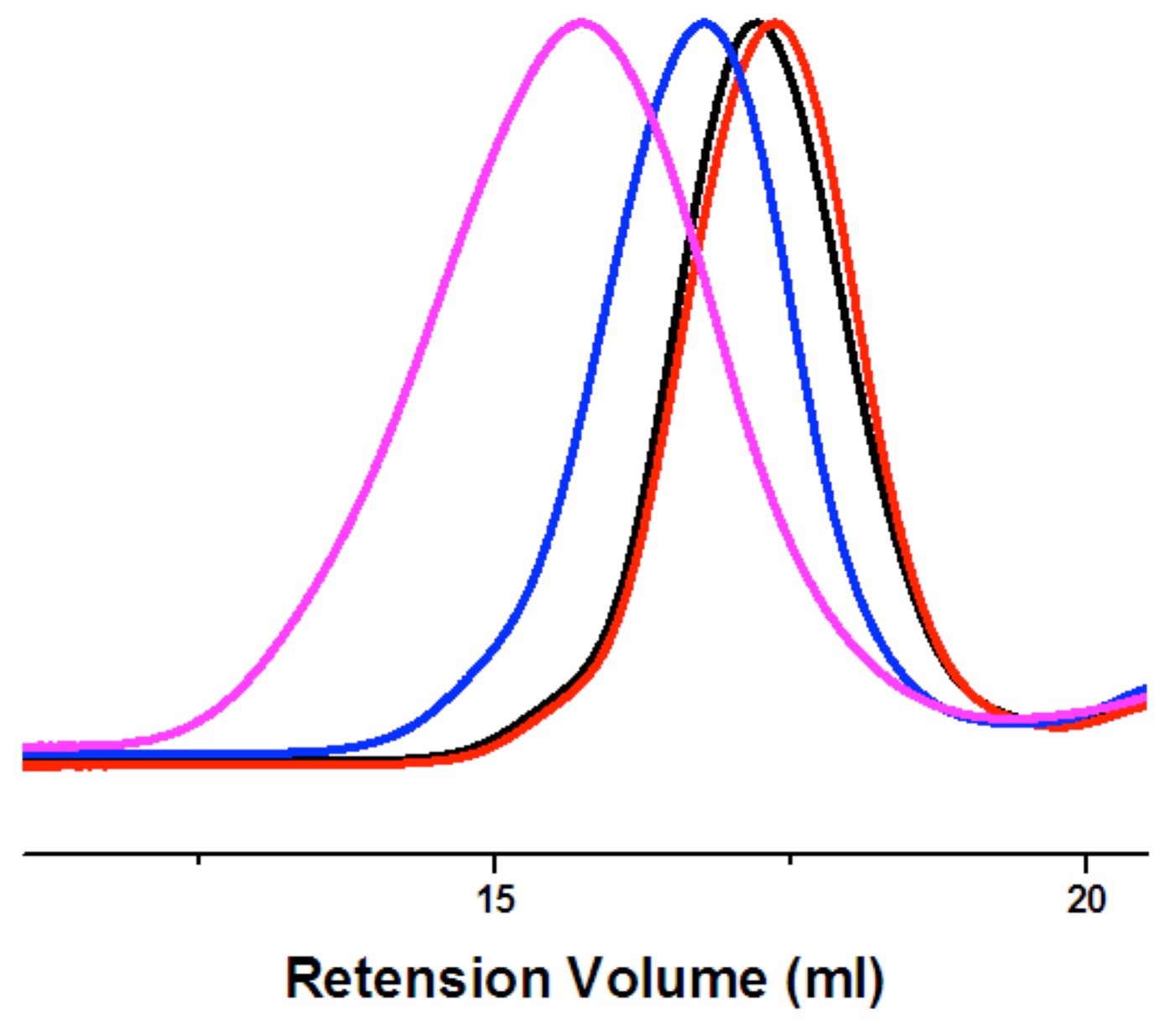


**Figure S1.** A) SEC chromatograms of macro-RAFT agents, from right to left: Qb-1 (red), Qb-2 (black), Qb-3 (blue) and Qb-4 (pink).

| (PEO MA)_X_-qb-(Sty)_Y_ | [Sty]/ [(PEO MA)_x_-CTA | PEO MA/  RAFT (NMR)^a^ | Sty/ RAFT  (NMR)^a^ | Conversion | | M_n_ _th_  (g mol^-1^)^b^ | Mn, SEC  (g mol^-1^)^c^ | Đ |
| --- | --- | --- | --- | --- | --- | --- | --- | --- |
|  |  |  |  | First Step | Second Step |  |  |  |
| **Qb-1** | 5 | 4.5 | 2.1 | 98.2 | 54.6 | 2879.5 | 2700 | 1.19 |
| **Qb-2** | 10 | 9.8 | 3.4 | 98.1 | 49.4 | 5461.6 | 4000 | 1.18 |
| **Qb-3** | 20 | 15.2 | 4.6 | 88.9 | 28.6 | 9368.5 | 5400 | 1.20 |
| **Qb-4** | 50 | 15.4 | 8.4 | 37.2 | 24.0 | 10416.1 | 8200 | 1.42 |

**Table** **S1.** RAFT synthesis of P(PEO MA)-qb-P(Sty)

^a^Determined by ^1^H NMR in DMSO-d6. ^b^
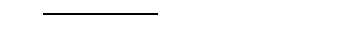
. ^c^Determined by SEC in THF (calibration Sty).

Table S2. Conditions used for the preparation of inverse HIPEs

| **Sample code** | **macro-RAFT agent** | **%wt** | **<D> (μm) Optical microscopy** | | **(SEM) (μm)** | |
| --- | --- | --- | --- | --- | --- | --- |
|  |  |  | Fresh | After 12h | <D> ^(1)^ | <d> ^(1)^ |
| **A1** | Qb-1 | 10 | 65.6 | 57.9 | - | - |
| **A2** | Qb-2 | 10 | 41.2 | 40.6 | - | - |
| **A3** | Qb-3 | 10 | 17.2 | 92.8 | - | - |
| **A4** | Qb-4 | 10 | - | - | - | - |
| **A5** | Qb-2 | 10 | 41.5 | 39.9 | 8.0 | 1.3 |
| **A6** | Qb-2 | 20 | 38.5 | 33.1 | 9.5 | 2.0 |
| **A7** | Qb-2 | 50 | - | - | - | 2.9 |
| **A8** | Qb-2 | 10 | 39.5 | 35.3 | 9.4 | 1.6 |
| **B1** | End group removed Qb-2 | 10 | 51.8 | 47.3 | 9.4 | 1.0 |

^(1)^ A void describes the pores of the PolyHIPE and <D> is average size of voids. Window refers to the interconnecting pores between two adjacent droplets and <d> is average size of windows.

**
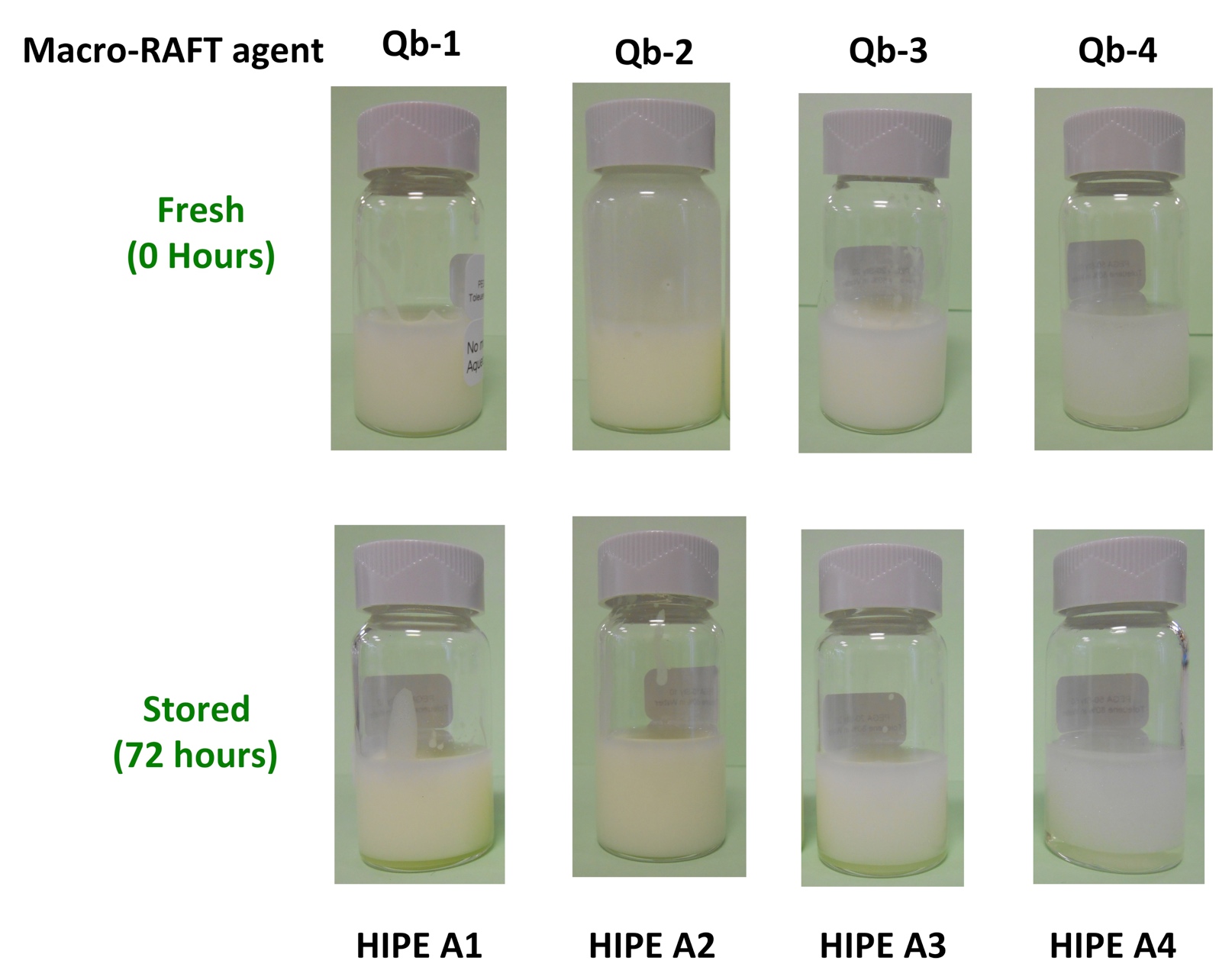
**

**Figure S2.** Stability of toluene in water HIPE stabilized by macro-RAFT agent. All HIPE with 80% toluene in water solely stabilized with 10 wt % of (from the left to right): macro-RAFT agent Qb1, macro-RAFT agent Qb2, macro-RAFT agent Qb3 and macro-RAFT agent Qb4. No monomers were introduced in the HIPEs.

**
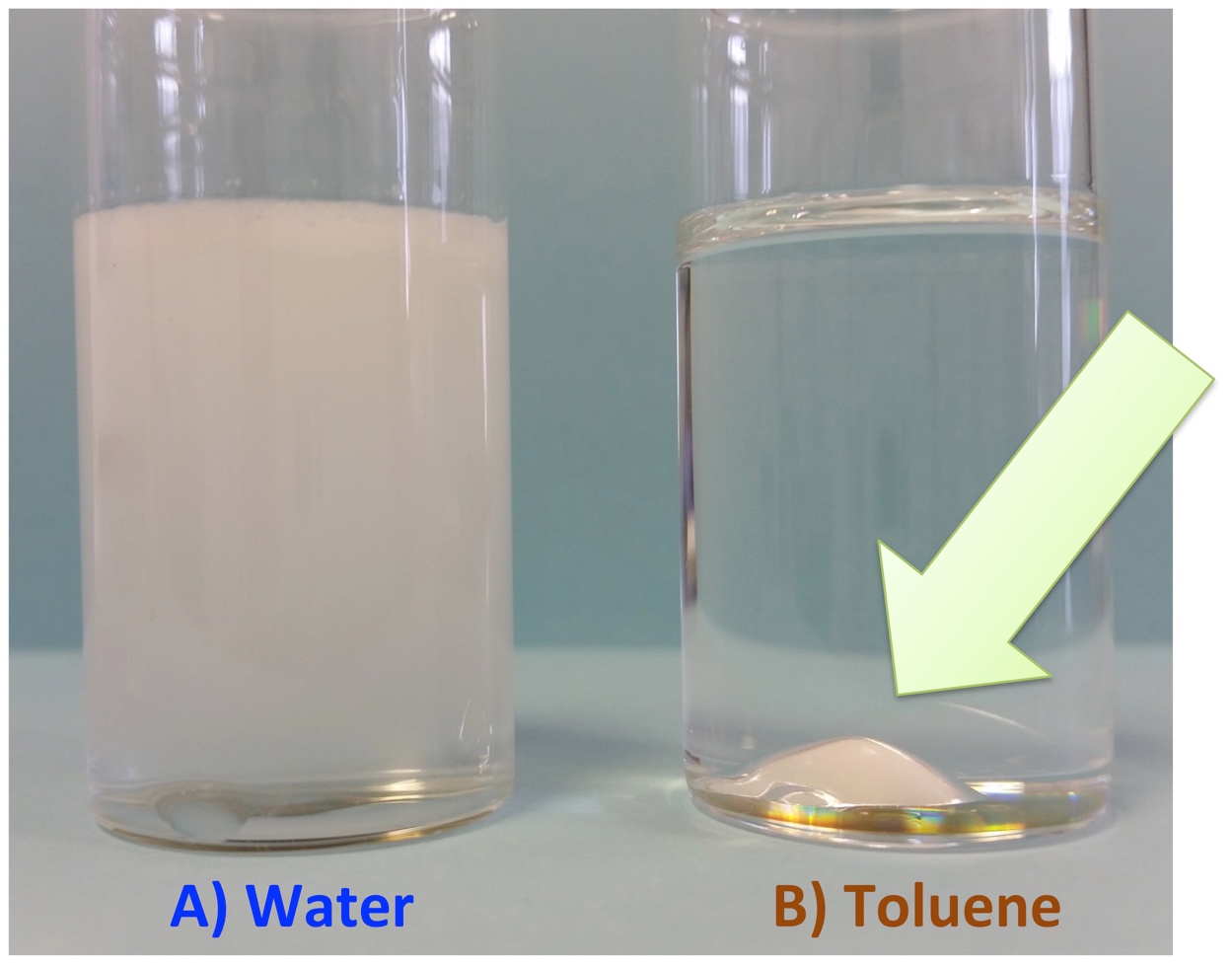
**

**Figure S3.** The emulsion type was determined by the drop test method. One drop of the formed HIPE with macro-RAFT agent-Qb2 was placed into (A) water and (B) toluene. The emulsion droplet was seen to disperse in the water but remained as a droplet in toluene.


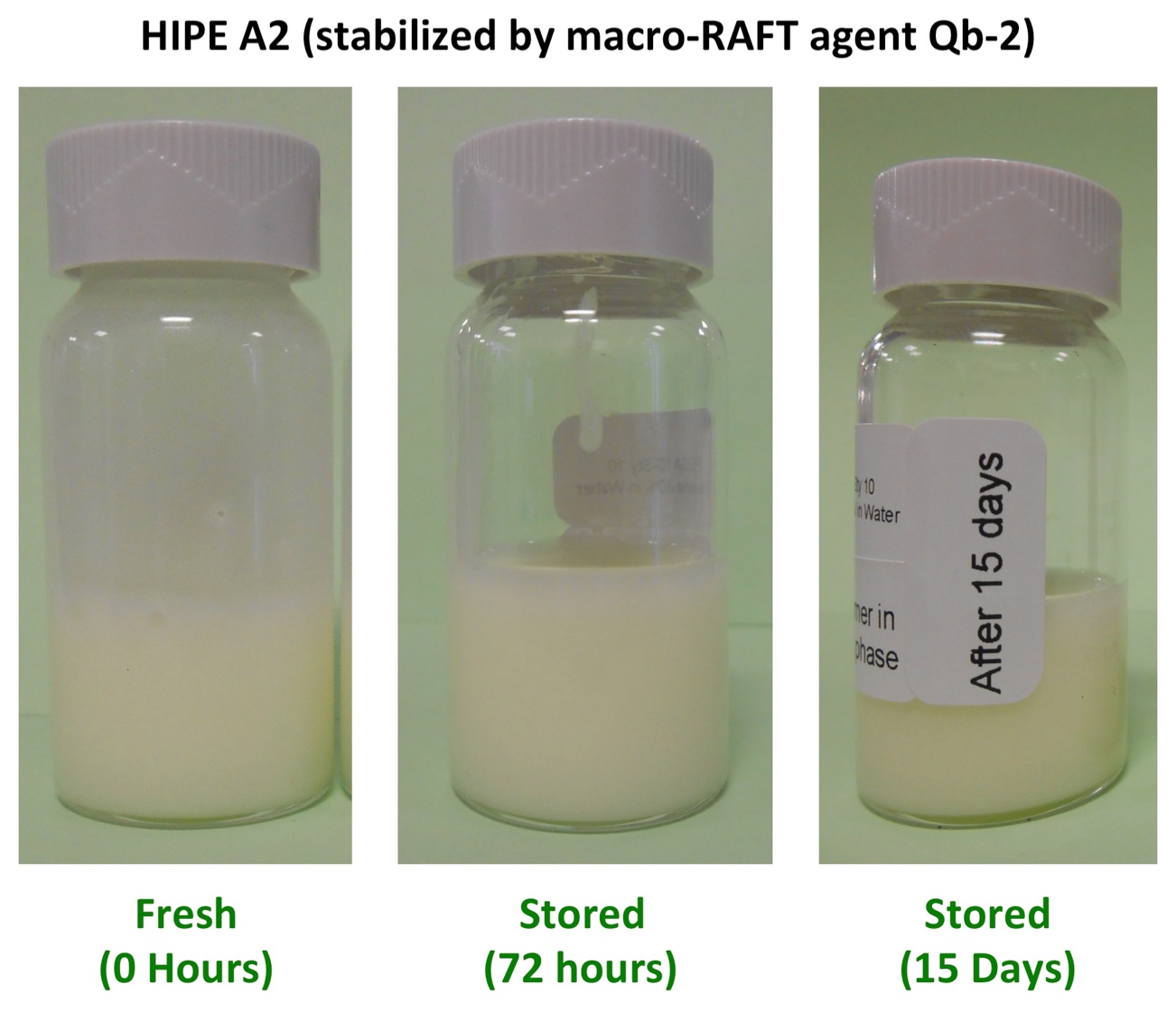


**Figure S4.** Stability of toluene in water HIPE stabilized by macro-RAFT agent-Qb-2.


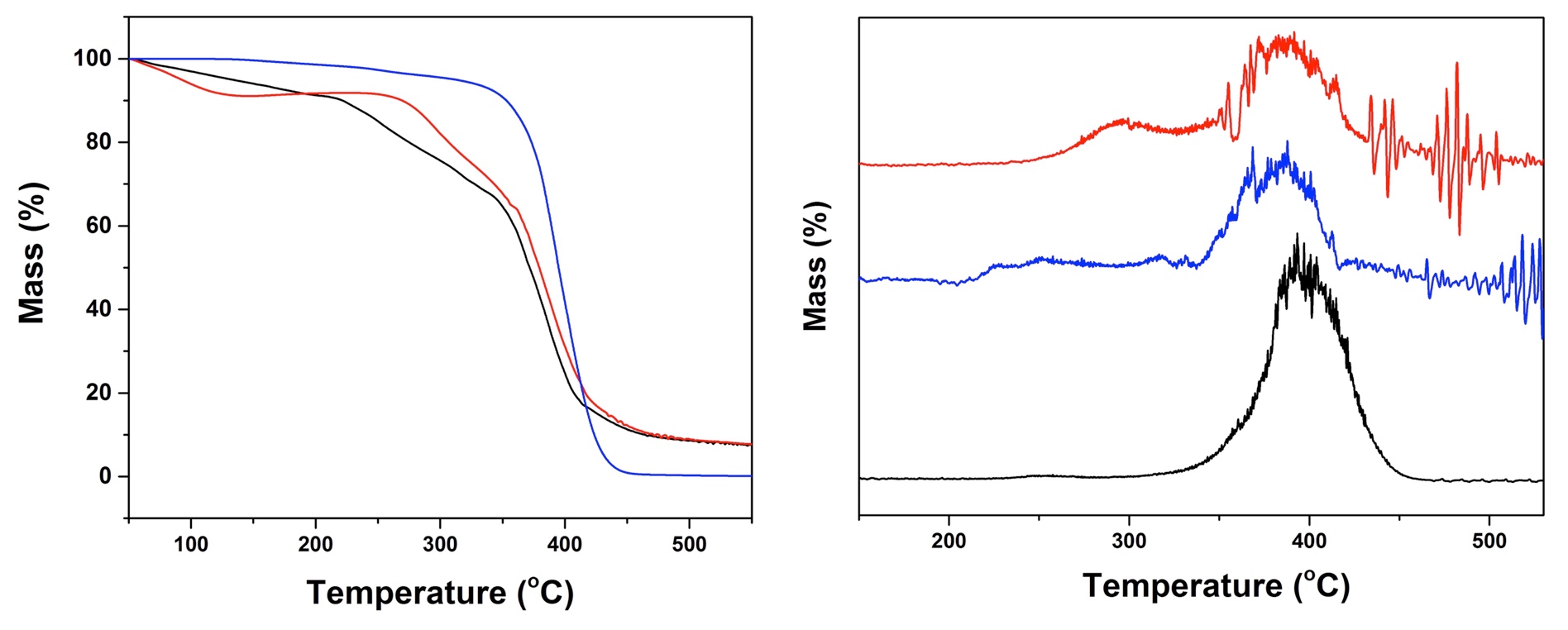


**Figure S5.** (A) TGA analysis of macro-RAFT agent-Qb2 (red), polyHIPE A5 (black) and bulk polymer (blue). B) T_max_ data.


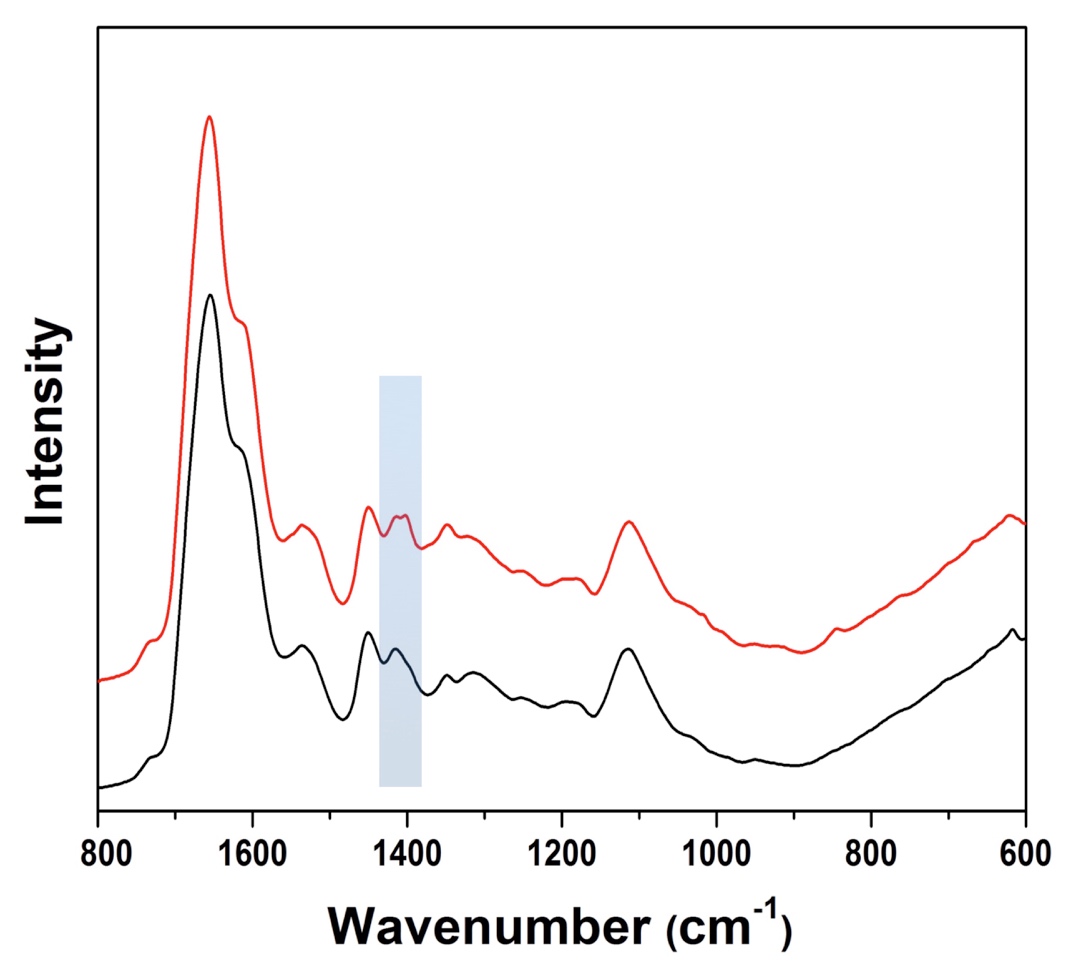


**Figure S6.** FTIR spectrum of poly(HIPE) A5 before (black) and after (red) “grafting from” polymerization of (4-vinylphenyl)boronic acid. The peak around 1375–1425 cm^−1^ is highlighted.


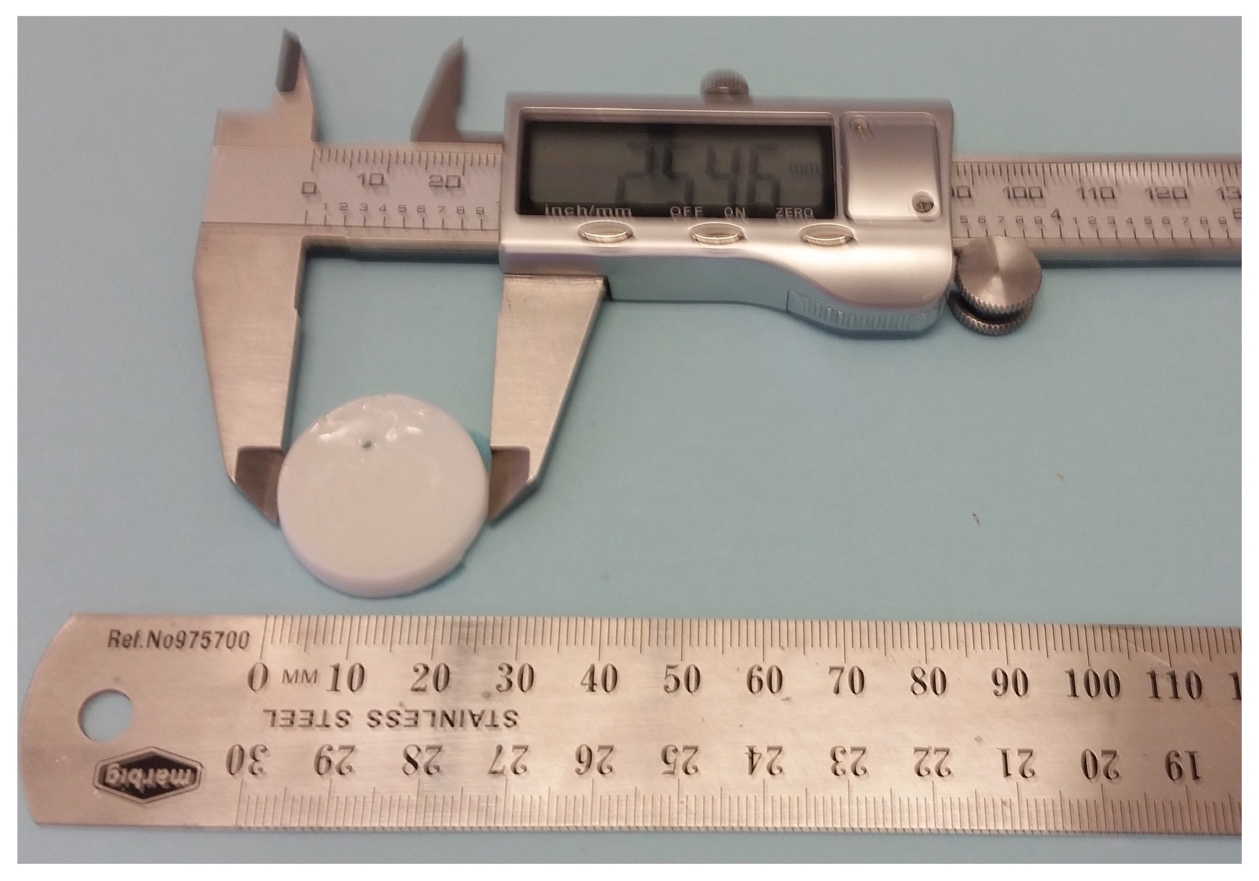


**Figure S7.** Shrinkage study on polyHIPE A5.


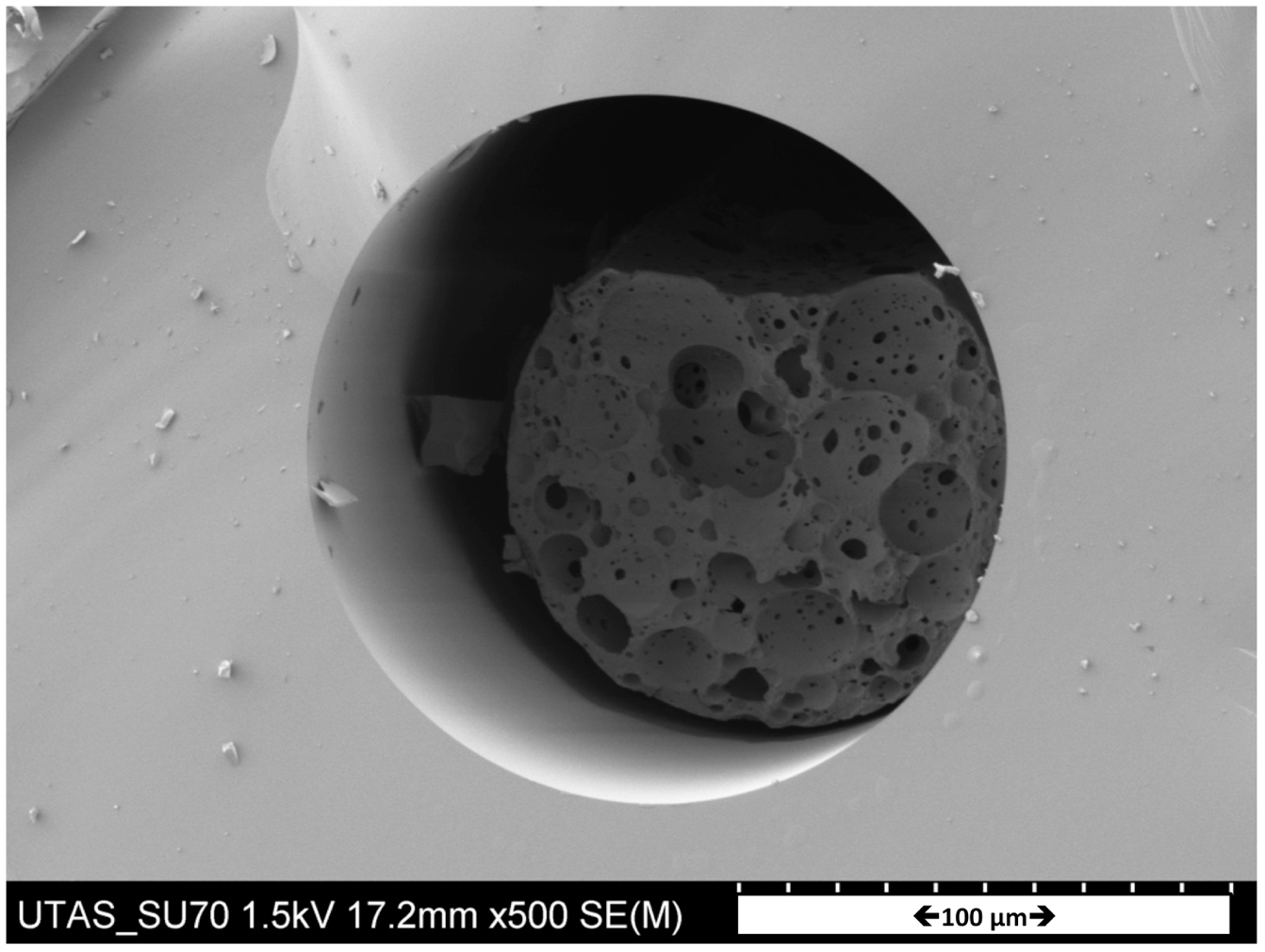


**Figure S8.** Polymerized HIPE B1 stabilized by end group removed macro-RAFT agent, *in situ* polymerization in 150 μm ID capillary.


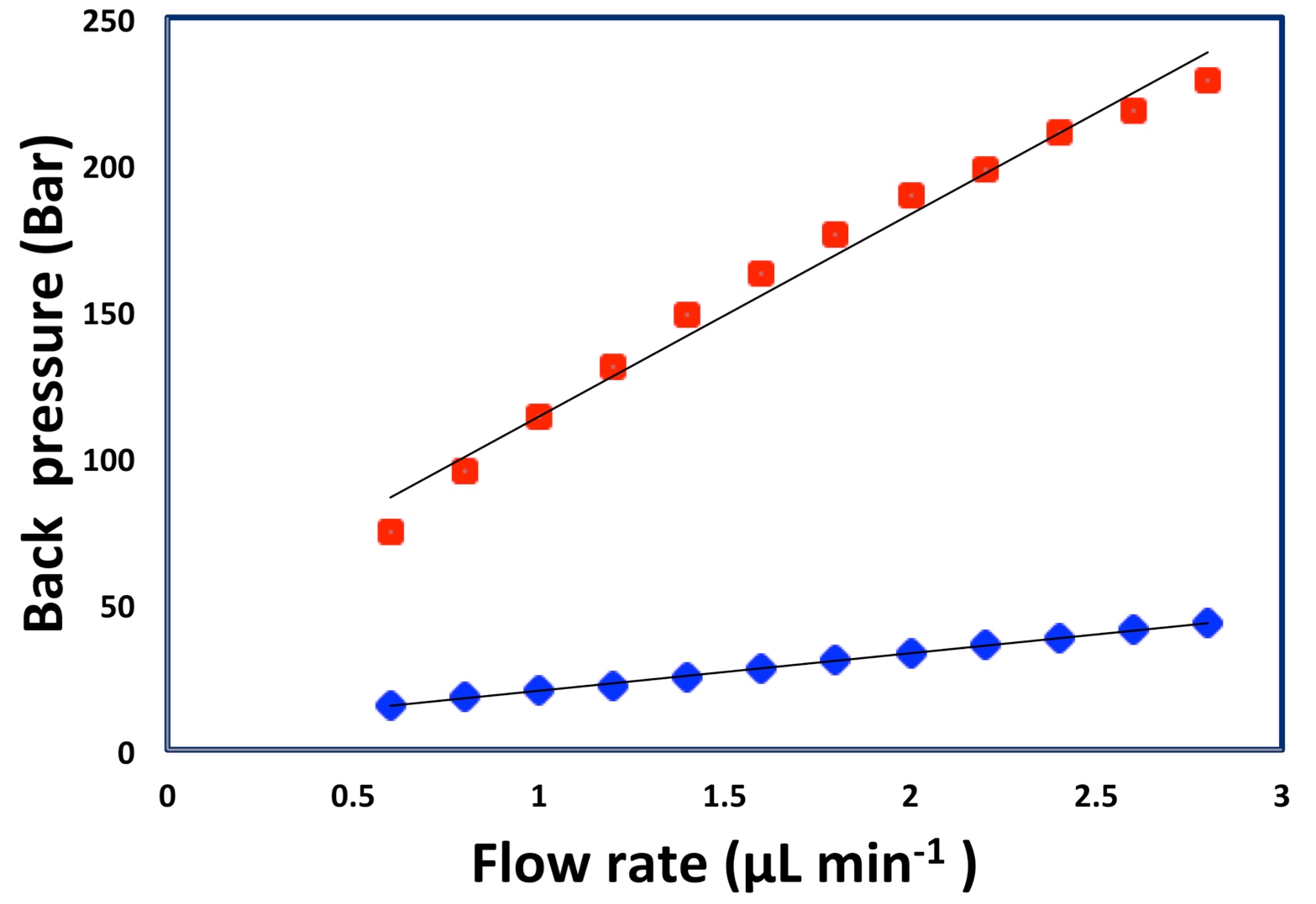


**Figure S9.** Back pressure studies on capillary A8, solvents MiliQ-water (red) and acetonitrile (blue).


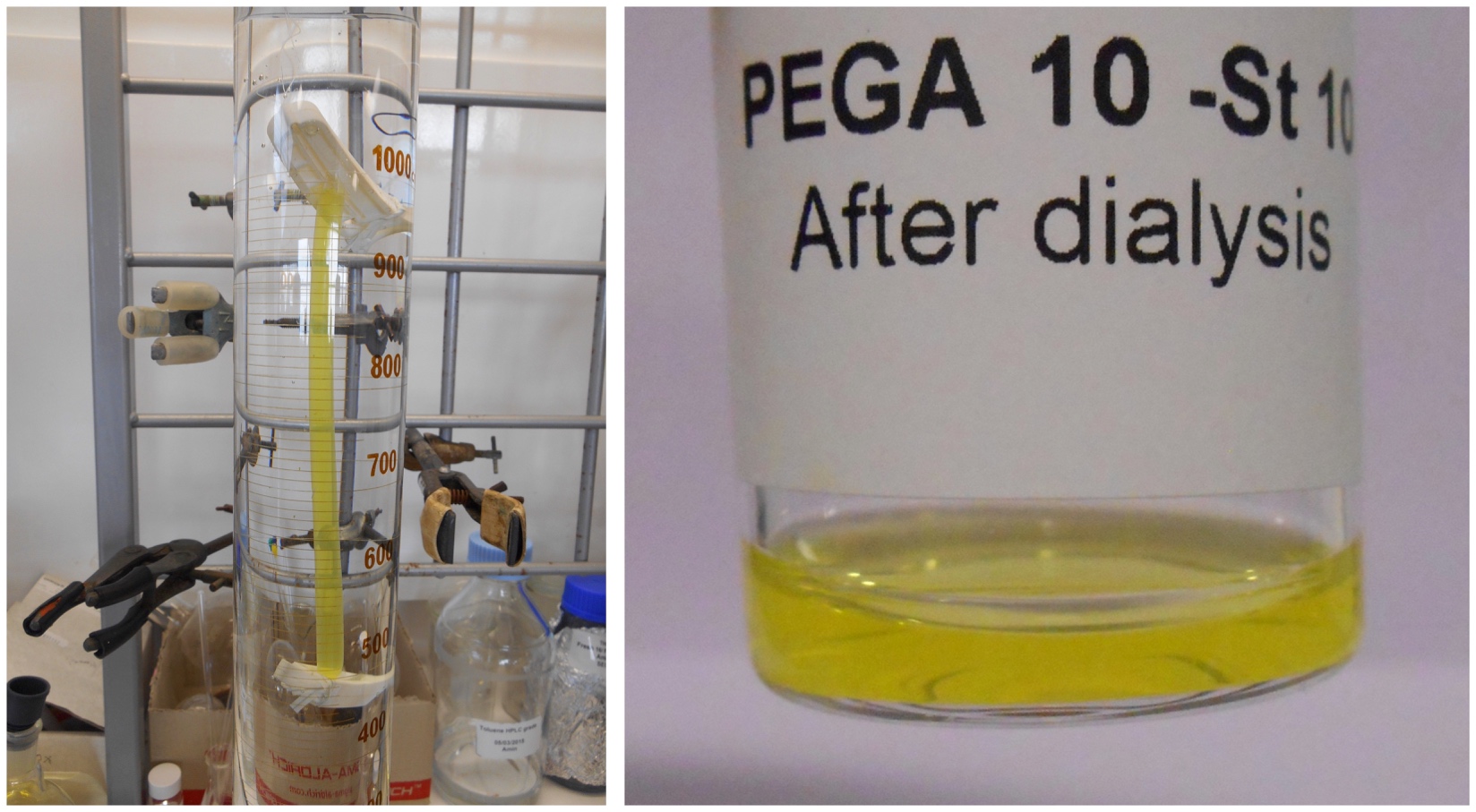


**Figure S10**. Typical procedure for purification of macro-RAFT agent by using dialysis tubing (MWCO 2000).


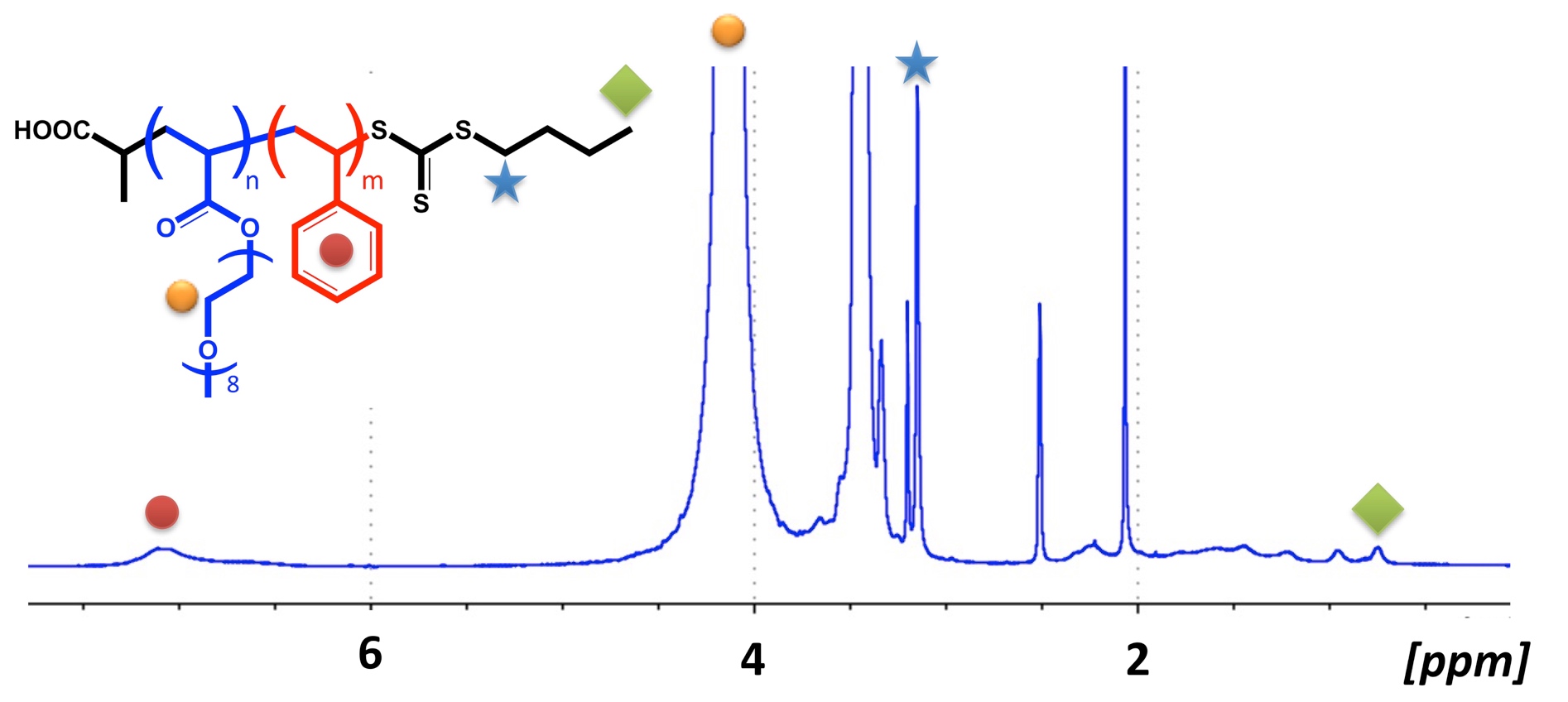


**Figure S11.** ^1^HNMR spectra of macro-RAFT agent Qb-2 (DMSO-d6).

**
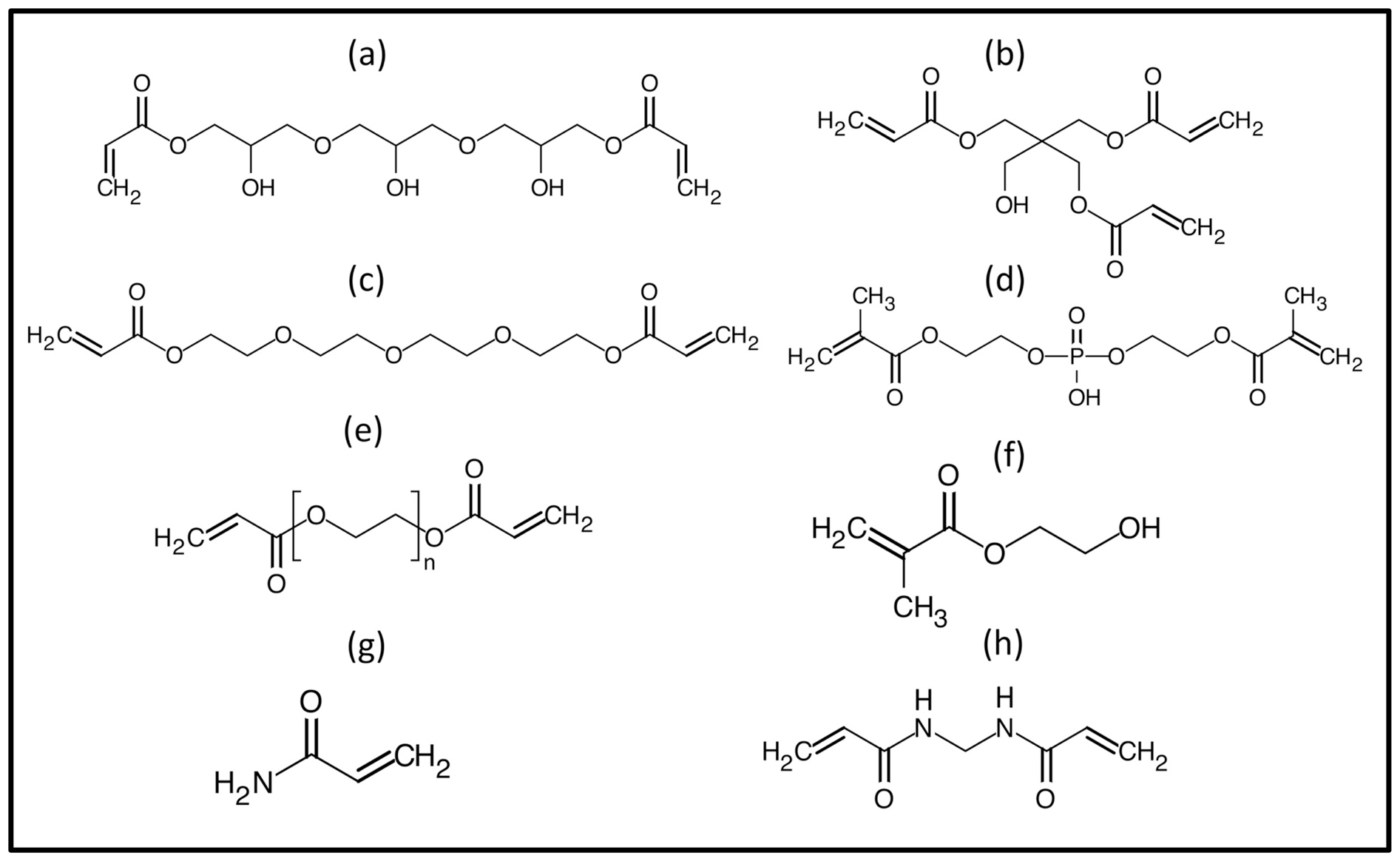
**

**Figure S12.** Monomers and cross-linkers used in aqueous phase.


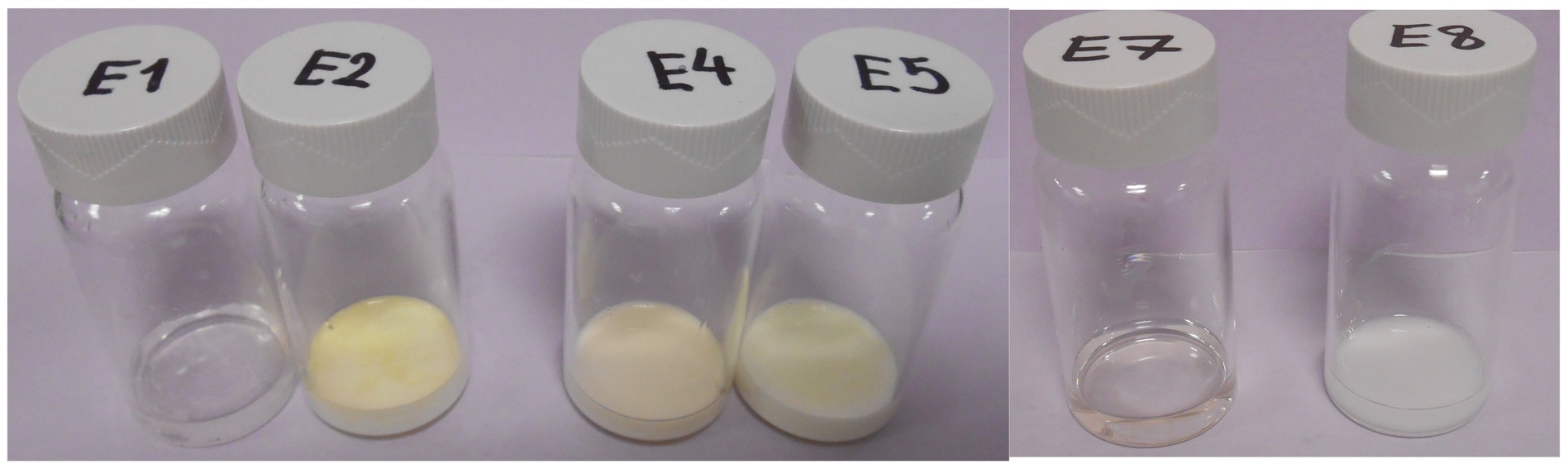


**Figure S13.** Typical polymerization of monomers in water in presence of redox initiation system (TEMED/ KPS). From the left to right: Glycerol 1,3-diglycerolate diacrylate, Pentaerythritol triacrylate, Tetra(ethylene glycol) diacrylate, Bis[2-(methacryloyloxy)ethyl] phosphate,  Poly (ethylene glycol) diacrylate, 2-Hydroxyethyl methacrylate, acrylamide.

**
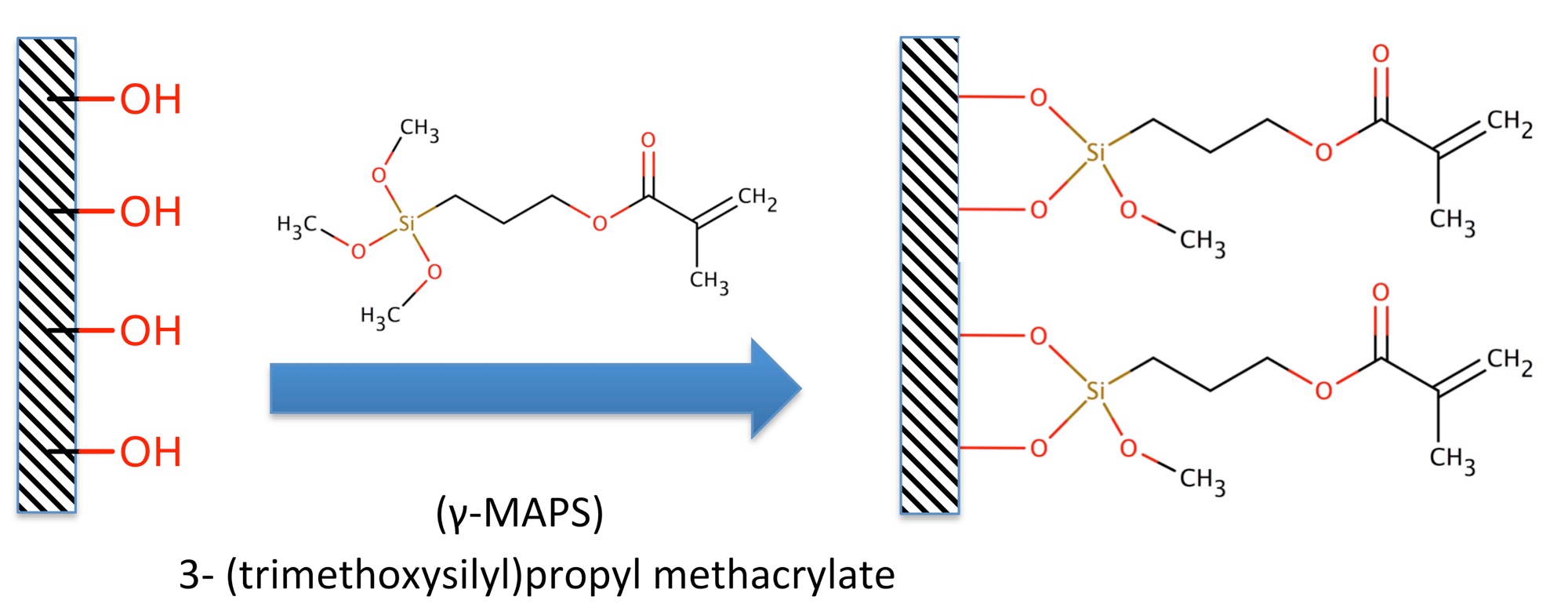
**

**Figure S14.** Surface modification of a fused-silica capillary surface using 3-(trimethoxysilyl)propyl methacrylate (γ -MAPS).

**
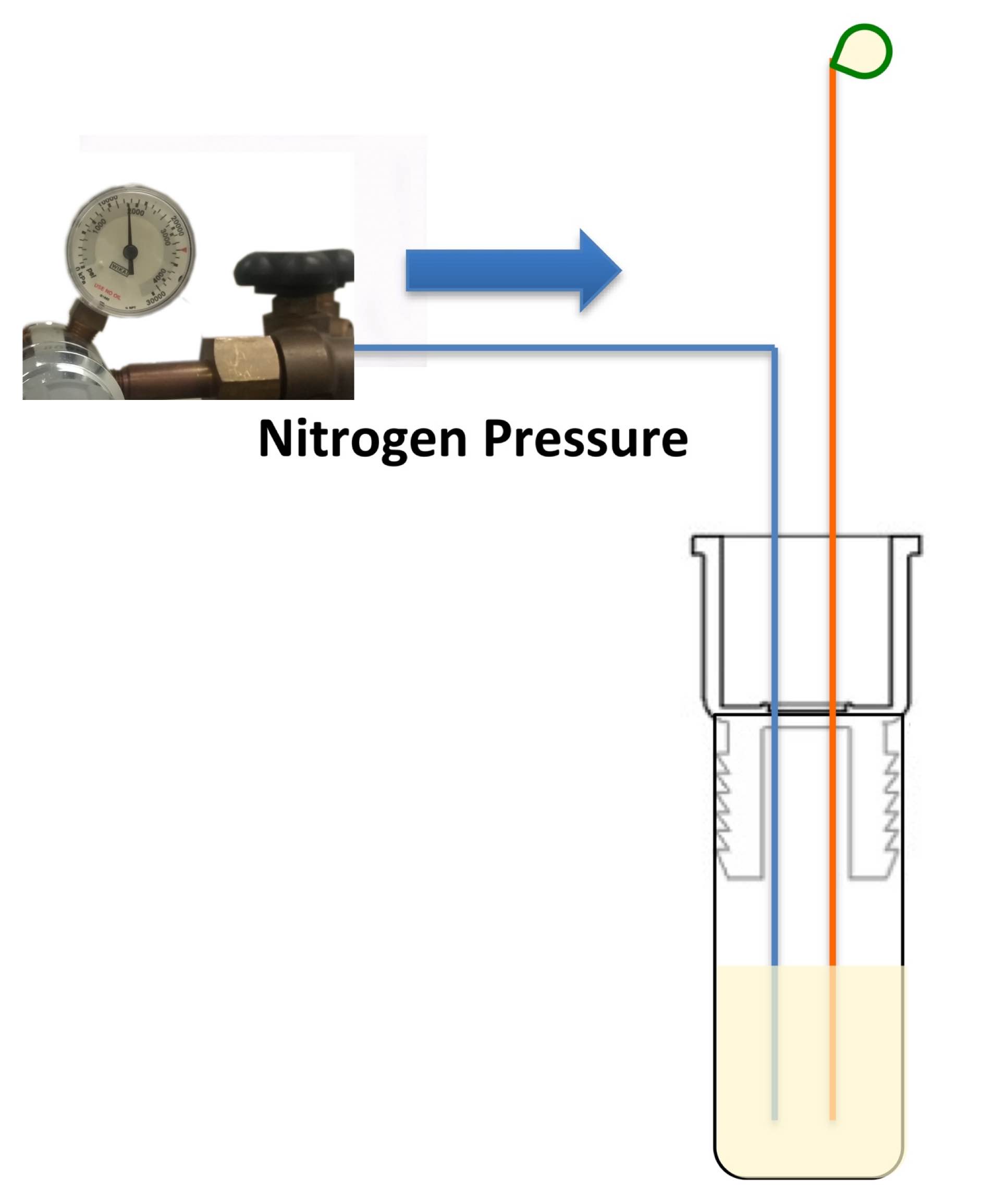
**

**Figure S15.** Using nitrogen pressure to fill a capillary format column with an inverse HIPE.
